# Integrative multi-omics and machine learning identify a robust signature for discriminating prognosis and therapeutic targets in bladder cancer

**DOI:** 10.7150/jca.105066

**Published:** 2025-01-27

**Authors:** Zhiyong Tan, Xiaorong Chen, Yinglong Huang, Shi Fu, Haihao Li, Chen Gong, Dihao Lv, Chadanfeng Yang, Jiansong Wang, Mingxia Ding, Haifeng Wang

**Affiliations:** 1 Department of Urology, The Second Affiliated Hospital of Kunming Medical University, No. 347, Dianmian Street, Wuhua District, Kunming, 650101, Yunnan, People's Republic of China.; 2 Urological disease clinical medical center of Yunnan province, The Second Affiliated Hospital of Kunming Medical University, No. 347, Dianmian Street, Wuhua District, Kunming, 650101, Yunnan, People's Republic of China.; 3 Scientific and Technological Innovation Team of Basic and Clinical Research of Bladder Cancer in Yunnan Universities, The Second Affiliated Hospital of Kunming Medical University, No. 347, Dianmian Street, Wuhua District, Kunming, 650101, Yunnan, People's Republic of China.; 4 Department of Kidney Transplantation, The Third Hospital of Sun Yat-Sen University, Guangzhou, People's Republic of China.

**Keywords:** Bladder cancer, Prognostic genes, Prognostic model, single-cell RNA sequencing

## Abstract

**Background:** Bladder cancer (BLCA) is a common malignant tumor whose pathogenesis has not yet been fully elucidated. This study analyzed prognostic genes in BLCA by integrating transcriptomics and proteomics data, and established prognostic models, aiming to offer novel insights for BLCA therapy.

**Methods:** Transcriptomic, proteomic, and protein acetylation sequencing were conducted on six BLCA tumor tissues and six paraneoplastic tissue samples. Furthermore, data from TCGA-BLCA, GSE13507, and single-cell RNA sequencing (scRNA-seq) datasets were integrated. Initially, differential expression analysis identified candidate genes regulated by acetylation. These genes were further refined by intersecting with scRNA-DEG obtained from the scRNA-seq dataset, resulting in the identification of key genes. Subsequently, consistency clustering analysis was performed based on these key genes. Prognostic models were then developed utilizing Cox regression analysis and least absolute shrinkage and selection operator (LASSO) Cox regression. Independent prognostic factors were determined through independent prognostic analysis, followed by the establishment of a nomogram model. Additionally, gene set enrichment analysis (GSEA), immune cell infiltration analysis, mutation analysis, and drug sensitivity analysis were conducted between the two risk groups to elucidate underlying mechanisms.

**Results:** A total of 15 key genes were obtained by crossing 284 candidate genes with 510 scRNA-DEGs. Patients in the TCGA-BLCA dataset were categorized into two subtypes based on the 15 key genes. Next, a risk model was developed using five prognostic genes (CTSE, XAGE2, MAP1A, CASQ2, and FXYD6), and a nomogram model was developed using age, pathologic T, pathologic N, and risk score. A total of 1089 GO entries and 49 KEGG pathways, including cytokine-cytokine receptor interactions, ECM receptor interactions, etc., were involved in all genes in both risk groups. The immunization score, matrix score, and ESTIMATE score were significantly higher in the low-risk group than in the high-risk group.

**Conclusion:** CTSE, XAGE2, MAP1A, CASQ2 and FXYD6 were selected as prognostic genes in BLCA, risk model and nomogram model predicting the prognosis of BLCA patients were constructed. These were helpful for prognostic assessment of BLCA.

## 1. Introduction

Bladder cancer (BLCA) is recognized for its pronounced heterogeneity in histological, molecular, and phenotypic dimensions^1^. It accounts for approximately 573,278 new cases and results in 212,536 deaths globally each year^2^, often presenting with advanced invasive and metastatic characteristics. Existing treatments for patients with muscle-invasive bladder cancer (MIBC) and metastatic BLCA primarily consist of integrative therapies, including chemotherapy, immunotherapy, and radical resection, but the development of recurrence and drug resistance often becomes obstacles for patients to achieve a favorable response and long-term survival^3^. Hence there is an imperative requirement to explore critical genes associated with BLCA and to generate efficacious prognostic models that can lead to personalized treatment of patients with BLCA.

With the evolution of sequencing technologies, a plethora of biomarkers related to the diagnosis and prognosis of BLCA have been identified at the transcriptomic and proteomic levels. Notably, biomarkers such as Apolipoprotein A1, MMP-1, Properdin, and Calgranulin B have been identified in the urine for diagnosis and staging of non-invasive BLCA^4^. Recent studies, including that by Bohyun Kim *et al.*, have demonstrated that tubulin beta 6 class V (TUBB6) harbors properties that promote the invasion and migration of BLCA cells, suggesting its potential role as a prognostic marker for BLCA patients^5^. Beyond traditional genomics and proteomics, novel assays are being developed to enhance the detection and monitoring of BLCA. For instance, the use of extracellular vesicle (EV)-based detection has shown promise, as EVs can carry tumor-specific proteins, lipids, and RNAs, enabling a non-invasive yet highly specific diagnostic approach. This method, which leverages advancements in EV isolation and molecular characterization, represents a step forward in improving early detection and disease monitoring^6^. In addition to traditional genomic and proteomic analyses, post-translational modification (PTM) has been gradually emphasized for its role in tumorigenesis and progression^7^. Acetylation, a vital PTM modification, is involved in the regulation of versatile biological processes such as glycolysis, lipid synthesis, DNA damage repair, and cell cycle^8^ and is an integral part of the development and progression of multiple tumors. Specifically, in BLCA, N-acetyltransferase 10 (NAT10) has been shown to facilitate BLCA cell proliferation, invasion, and migration by regulating the N4-acetylcysteine modification process of mRNAs, including BCL9L, SOX4, and AKT1^9^. Moreover, research by Yan Sun *et al.*, has revealed that silencing SIRT1 increased the Beclin1 acetylation, suppressing autophagy and decreasing cisplatin resistance^10^. Such findings underscore the paramount importance of understanding the regulatory mechanisms mediated by protein acetylation. Therefore, in-depth excavation of genes regulated by protein acetylation would contribute to unraveling the complex intracellular regulatory mechanisms and open up exhilarating avenues for therapeutic interventions in BLCA.

Conventional molecular biology techniques, including genomics, transcriptomics, and proteomics, are limited in their ability to fully capture the complex characteristics of BLCA. These methods often overlook the tumor microenvironment, cellular heterogeneity, and tumor-host immune interactions. In the present study, we collected 6 BLCA tumor tissues and 6 paracancerous tissue samples, performed transcriptomics, proteomics, and protein acetylation sequencing, and combined with single-cell RNA-seq to conduct a multidimensional comprehensive analysis. This approach enables us to identify acetylation genes and crucial cell subtypes related to the prognosis of BLCA patients and to establish a prognostic model. Accordingly, BLCA patients were divided into high and low-risk groups, and we observed differences in clinicopathological features, signaling pathways, mutational landscape, and immune cell infiltration among the two groups. Furthermore, our results strongly conjectured that there were also discrepancies in therapeutic response across the two groups, which was confirmed by drug prediction at the end of our study. To summarize, by cross-validating and complementing data from multiple sequencing technologies, we enhance interpretation accuracy and reliability, laying the groundwork for precision medicine research and clinical applications in BLCA, ultimately promoting individualized medical practices and improving patient outcomes.

## 2. Results

### 2.1 Identification of DEGs, DEPs and DEPAs

**Figure 1:**
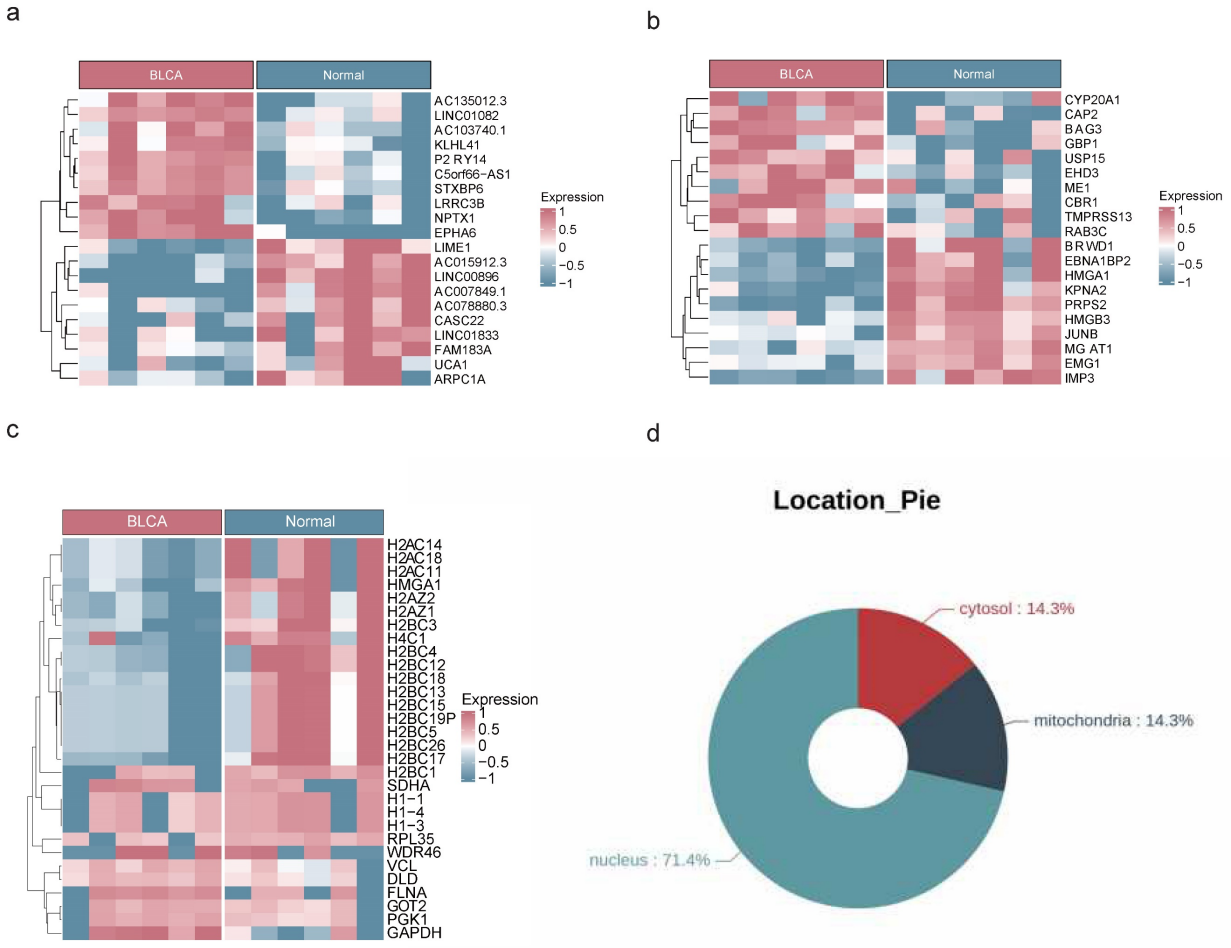
** Acquisition of DEGs, DEPs and DEPAs.** Heat maps of DEGs **(a)**, DEPs **(b)** and DEPAs **(c)** between BLCA and controls in transcriptomic sequencing dataset, proteomics dataset and acetylomics dataset, respectively. **(d)** The circle diagram showing the subcellular localization of DEPAs.

The analysis of transcriptome, proteome, and protein acetylation sequencing data revealed marked disparities between BLCA tumor samples and their normal counterparts, without any detectable outliers (**Figure S1a-c**). The transcriptomic landscape uncovered a total of 4,821 DEGs with 3,001 being upregulated and 1,820 downregulated in the BLCA samples (**Figure 1a, Figure S2a**). Proteomic profiling identified 2,395 DEPs, among which 1,860 exhibited increased expression and 535 showed decreased expression in BLCA samples (**Figure 1b, Figure S2b**). Additionally, comprehensive protein acetylation sequencing revealed 30 DEPAs, with an upward trend seen in 22 and a downward trend in 8 (**Figure 1c**). Enrichment analysis revealed that DEGs and DEPs were involved in the calcium ion signaling pathway, Apelin signaling pathway, and Wnt signaling pathway, while the functions of DEPAs were mainly related to neutrophil extracellular trap formation, glycolysis necrotic apoptosis, and so on (**Figure S3a-f**). In addition, subcellular localization analysis revealed that 71.4% of DEPAs might hold significant roles in the nucleus (**Figure 1d**).

### 2.2 A sum of 284 candidate genes were regulated by acetylation

**Figure 2:**
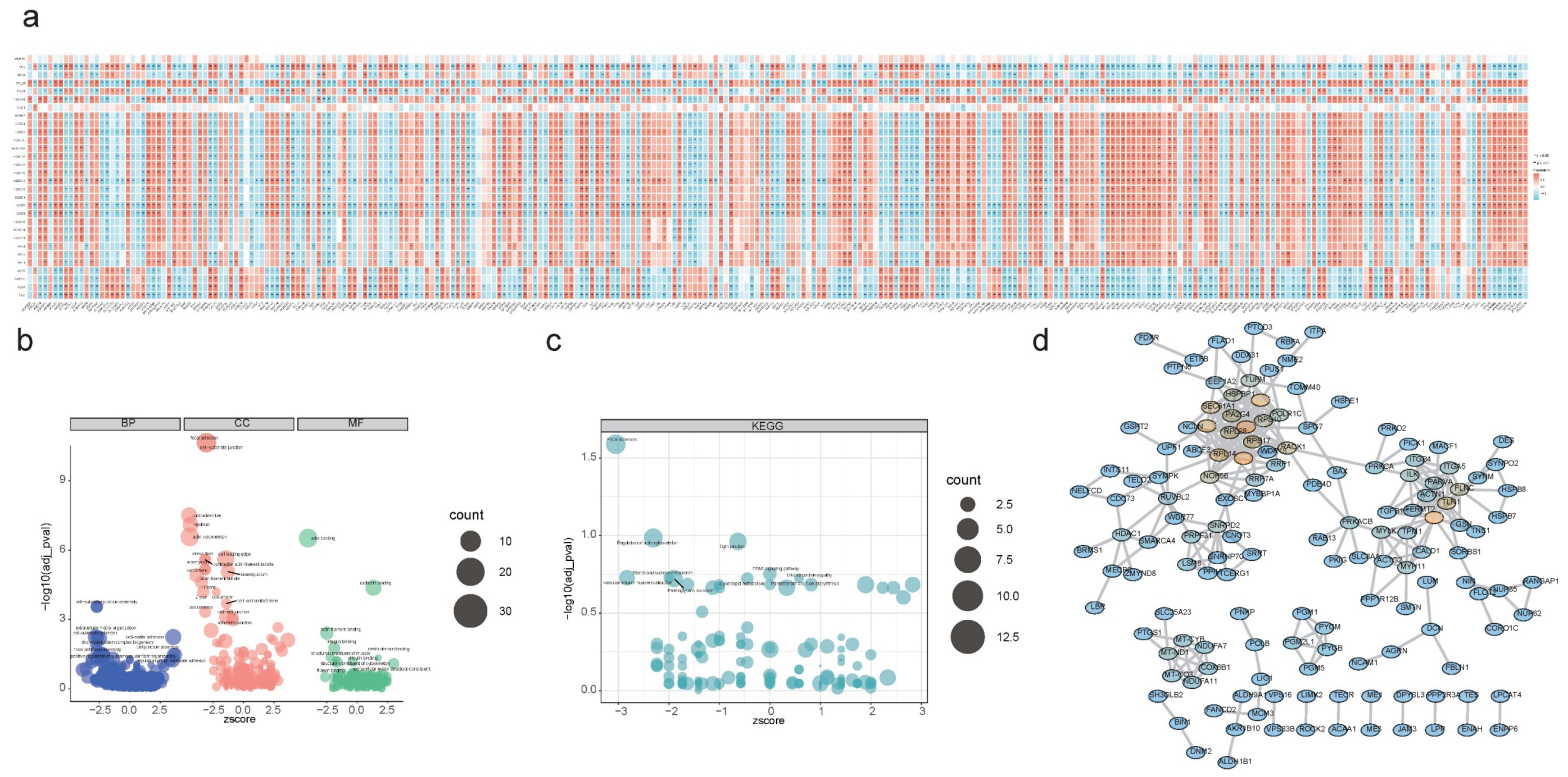
** Functional enrichment analysis of candidate genes. (a)** Spearman's correlation analysis between DEGs/DEPs and DEPAs, red indicates positive correlation, blue indicates negative correlation. (* P < 0.05, ** P < 0.01, *** P < 0.001, **** P < 0.0001). **(b)** GO enrichment analysis of candidate genes. **(c)** KEGG enrichment analysis of candidate genes. **(d)** PPI network exhibiting the close interactions among candidate genes.

An overlapping analysis of DEGs and DEPs pinpointed 291 intersection genes/intersection proteins sharing the same expression trend, comprising 119 down-regulated and 172 up-regulated candidate genes (**Figure S4a-b**). Spearman correlation analysis revealed 284 PA-DEGs likely modulated by acetylation, which predominantly contribute to the regulation of the central PPAR signaling pathway, actin cytoskeleton organization, and glycine metabolism, among others (**Figure 2a-c**). Subsequent PPI analysis demonstrated significant interactions among these genes, exemplified by pairs such as PRKACB-MYLK, VCL-TLN1, and TOMM40-TUF (**Figure 2d**).

### 2.3 Totally 13 cell clusters were merged into 5 cell types

**Figure 3:**
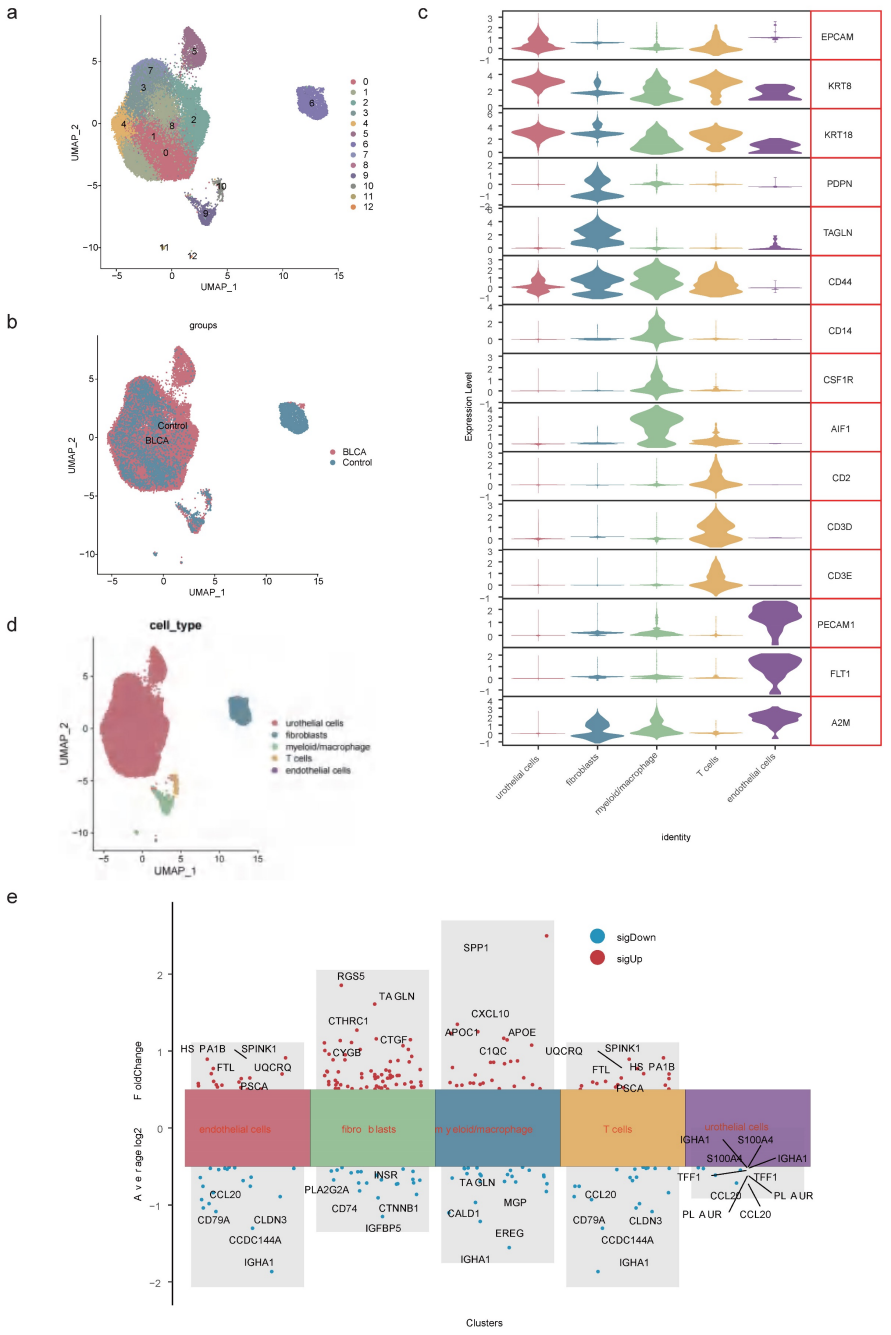
** Identification of cell types. (a)** UMAP dimensionality reduction of marker genes in the samples. **(b)** Distribution of cell clusters in BLCA tumor tissue and controls. **(c)** Expression patterns of marker genes in 5 cell types. **(d)** Distribution of urothelial cells, fibroblasts, myeloid macrophages, T cells and endothelial cells. **(e)** Expression levels of scRNA-DEGs between discrepant cell types.

After data processing for the single-cell dataset GSE135337, Figure S5a-b displayed the number of nFeature RNA and nCount RNA (**Table 1**). A total of 2,000 hypervariable genes were highlighted with blue marks in Figure S6. The top 30 principal components were selected through PCA, as shown in **Figure S5c-d**. UMAP dimensionality reduction revealed 13 distinct cell clusters (**Figure 3a-b**), which were further categorized into 5 cell types based on the expression of marker genes. These cell types included urothelial cells, fibroblasts, myeloid macrophages, T cells, and endothelial cells (**Figure 3c-d, Table 2**). Notably, in GSM5329919, fibroblasts, and myeloid macrophages they exhibited a higher proportion, while urothelial cells were predominant in BLCA tumor tissues compared to controls (**Figure S5e-f**). Furthermore, the expression levels of 510 scRNA-DEGs in various cell types were presented in **Figure 3e.** For instance, IGHAI, S100A4 and TFF1 were down-regulated in urothelial cells.

### 2.4 Identification and functional network of key genes

**Figure 4:**
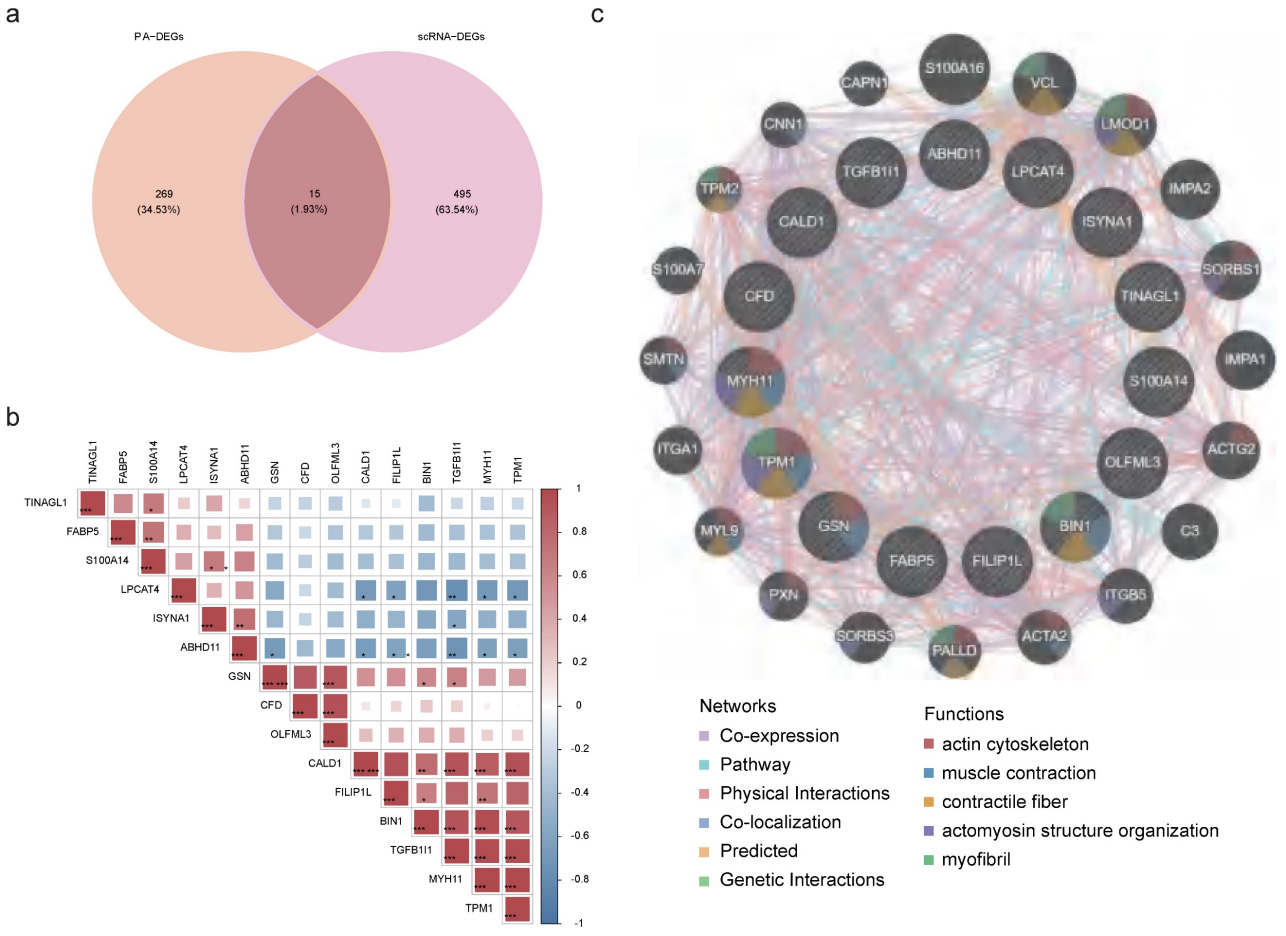
** Correlation between key genes. (a)** Venn diagram of 284 candidate genes and 510 scRNA-DEGs intersections. **(b)** Heatmap of correlations between key genes. **(c)** GGI network exhibiting interactions between pivotal genes and 20 other genes.

Then, through an intersection analysis of 284 candidate genes and 510 scRNA-DEGs, 15 key genes were identified (ABHD11, BIN1, CALD1, CFD, FABP5, FILIP1L, GSN, ISYNA1, LPCAT4, MYH11, OLFML3, S100A14, TGFB1I1, TINAGL1, and TPM1) (**Figure 4a**). Notably, FABP5 exhibited a positive correlation with S10OA104, while TGFB1I1 displayed negative correlations with ABHD11, ISYNA1, and LPCAT4 (**Figure S7, Figure 4b**). Additionally, a GGI network was constructed to illustrate interactions among these key genes and 20 others sharing similar biological functions. These intricate interactions were observed to involve processes such as muscle contraction, actin cytoskeleton regulation, and myofibril organization (**Figure 4c**).

### 2.5 Fibroblasts and myeloid macrophages were crucial cell types

**Figure 5:**
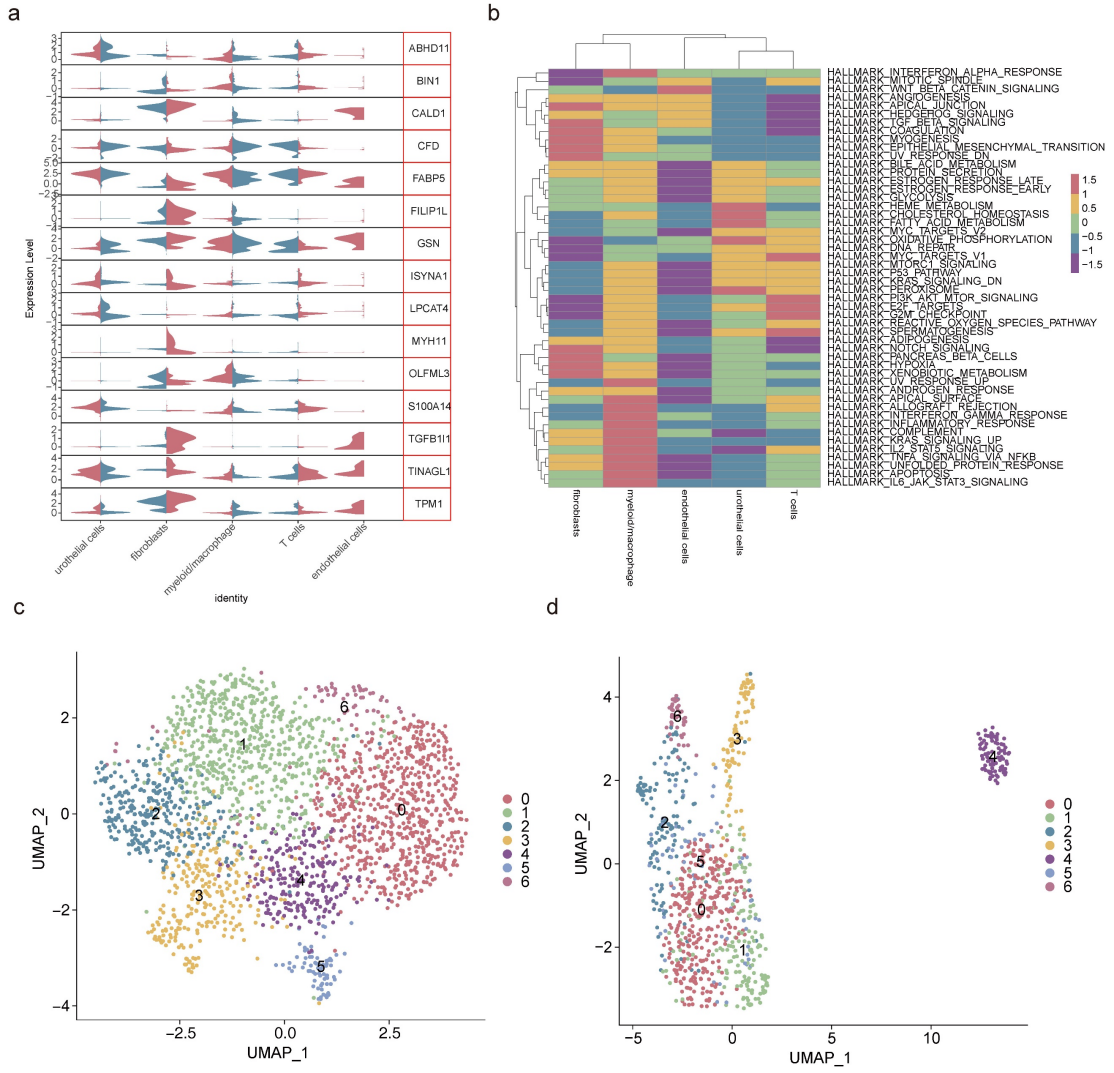
** Screening of crucial cell types. (a)** Violin plot of key genes' expression in 5 cell types. **(b)** Gene set variation analysis of 5 cell types. Secondary degradation and clustering of fibroblasts **(c)** and myeloid macrophages** (d)**.

Analysis of the expression patterns of key genes across 5 cell types revealed fibroblasts and myeloid macrophages as pivotal due to their disparate gene expression profiles (**Figure 5a, Table 3**). Pathway enrichment analysis highlighted fibroblasts' positive correlation with pathways such as coagulation and myogenesis, while myeloid macrophages exhibited negative correlations with the Wnt/β-catenin pathway and oxidative phosphorylation (**Figure 5b**). To elucidate the heterogeneity within these crucial cell types, secondary dimensionality reduction and clustering techniques were employed, resulting in the subdivision of fibroblasts and myeloid macrophages into 7 distinct subclusters each (**Figure 5c-d, Figure S8a-b**). Notably, subcluster 3 and subcluster 5 of fibroblasts, as well as subcluster 0 and subcluster 2 of myeloid macrophages, were found to be prevalent in BLCA, while other subclusters exhibited lower proportions in this context (**Figure S8c-d**).

### 2.6 Differential cellular dynamics and communication in BLCA

**Figure 6:**
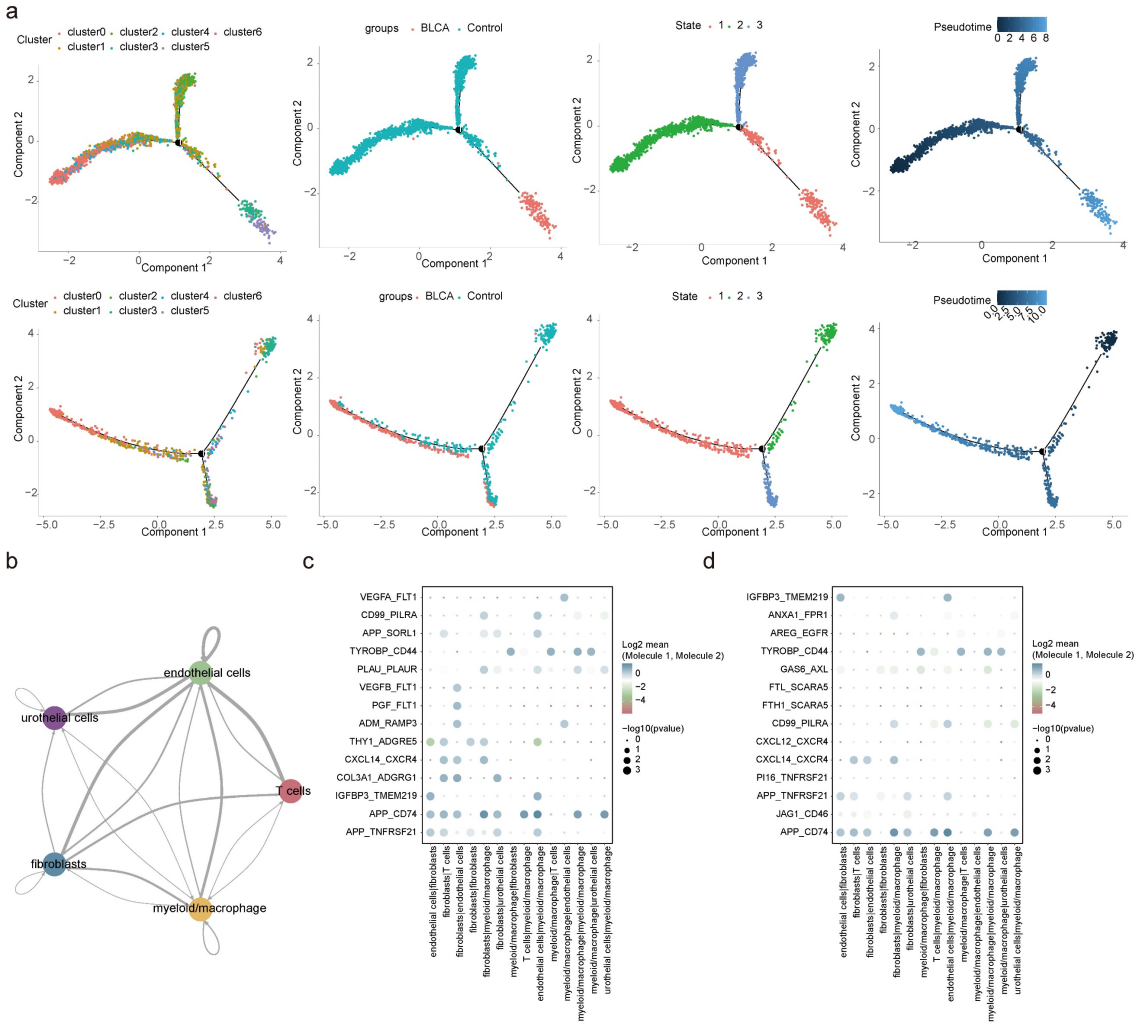
** Pseudo-temporal trajectory and cell communication analyses. (a)** Pseudo-temporal trajectory of fibroblasts and myeloid macrophages. **(b)** Interaction numbers between 5 cell types. Bubble plots presenting specific ligand-receptor pairs for intracellular signaling in BLCA **(c)** and controls **(d)**.

Pseudo-temporal analysis revealed that fibroblasts and myeloid macrophages underwent a differentiation process that could be categorized into three distinct stages (state 1, 2, and 3). It was observed that in BLCA, fibroblasts exhibited a more advanced differentiation state compared to non-cancerous controls. Furthermore, the composition of fibroblasts and myeloid macrophages diverged substantially at stage 3 when comparing BLCA tumor tissues with non-tumorous controls (**Figure 6a**). Corresponding to the progression of fibroblast differentiation, the expression levels of BIN1, GSN, OLFML3, and CFD were found to decline, whereas the expression of other pivotal genes generally increased, apart from S100A14 and LPCAT4, which remained constant (**Figure S9a**). For myeloid macrophages, an increase in differentiation was associated with reduced expression levels of CALD1, CFD, and TPM1 (**Figure S9b**).

An in-depth exploration of cellular communication revealed intricate interaction patterns among five cell types, facilitated through ligand-receptor engagement. The interactions between fibroblasts and endothelial cells, as well as between fibroblasts and myeloid macrophages, featured the most extensive network of receptor-ligand pairs, with the highest counts as shown in **Figure 6b and Figure S9c**. A curated selection of ligand-receptor pairs was portrayed in **Figures 6c-d**, revealing a subset shared between BLCA and control environments—specifically, interaction pairs such as APP-TNFRSF21, APP-CD74, IGFBP3-TMEM219, CXCL14-CXCR4, TYROBP-CD44, and CD99-PILRA stood out, illustrating the conservation of certain cellular communication pathways despite the diseased state.

### 2.7 A sum of 318 candidate prognostic genes was searched in the TCGA-BLCA dataset

**Figure 7:**
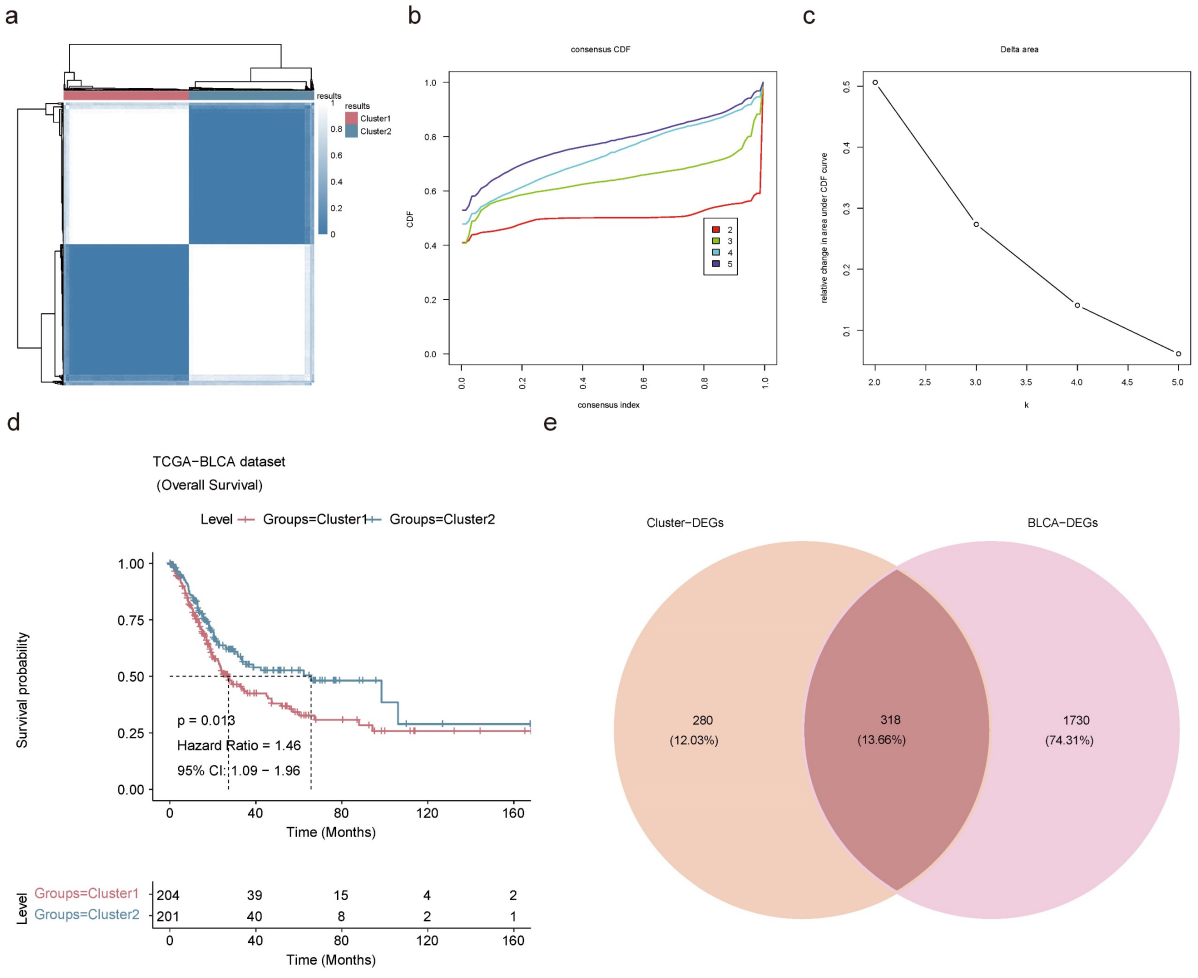
** Identification of BLCA-related subtypes. (a)** Consensus clustering of BLCA patients based on the expression of pivotal genes when k = 2. **(b)** CDF curve of K = 2-5. **(c)** The relative change in area under the CDF curve of K = 2-5. **(d)** K-M curve for overall survival of discrepant clusters via Log-rank test. **(e)** Venn diagram of intersection of Cluster-DEGs and BLCA-DEGs to select candidate prognostic genes.

Based on the 15 key genes from the above results, unsupervised cluster analysis was performed on TCGA-BLCA patients with 100 clusters, and BLCA patients were classified into 2 subtypes according to the CDF curve with K=2. This decision was grounded in an analysis of K-M survival estimates and the evaluation of relative changes in the area under the CDF curve across different k values (**Figure 7a-c**). Through cluster analysis, differences in survival rates between different subtypes could be identified, which could help to better predict the prognosis of BLCA patients. K-M survival analysis exposed a grim prognosis for patients categorized within Cluster 1 (**Figure 7d**). A differential expression analysis was carried out on the cohort comprising 204 patients from Cluster 1 and 201 from Cluster 2-unearthed 562 up-regulated and 36 down-regulated Cluster-DEGs (**Figure S10a-b**). The Cluster-DEGs were linked with latent functions related to protein digestion and absorption, PI3K-Akt signaling pathway, ECM-receptor interaction, and other functions (**Figure S10c-d**). There were 1,131 up-regulated BLCA-DEGs and 917 down-regulated BLCA-DEGs in the TCGA-BLCA dataset (**Figure S10e-f**). A sum of 318 candidate prognostic genes was selected by intersecting 598 Cluster-DEGs with 2,048 BLCA-DEGs for screening of prognostic genes (**Figure 7e**).

### 2.8 Establishment and validation of the risk model

**Figure 8:**
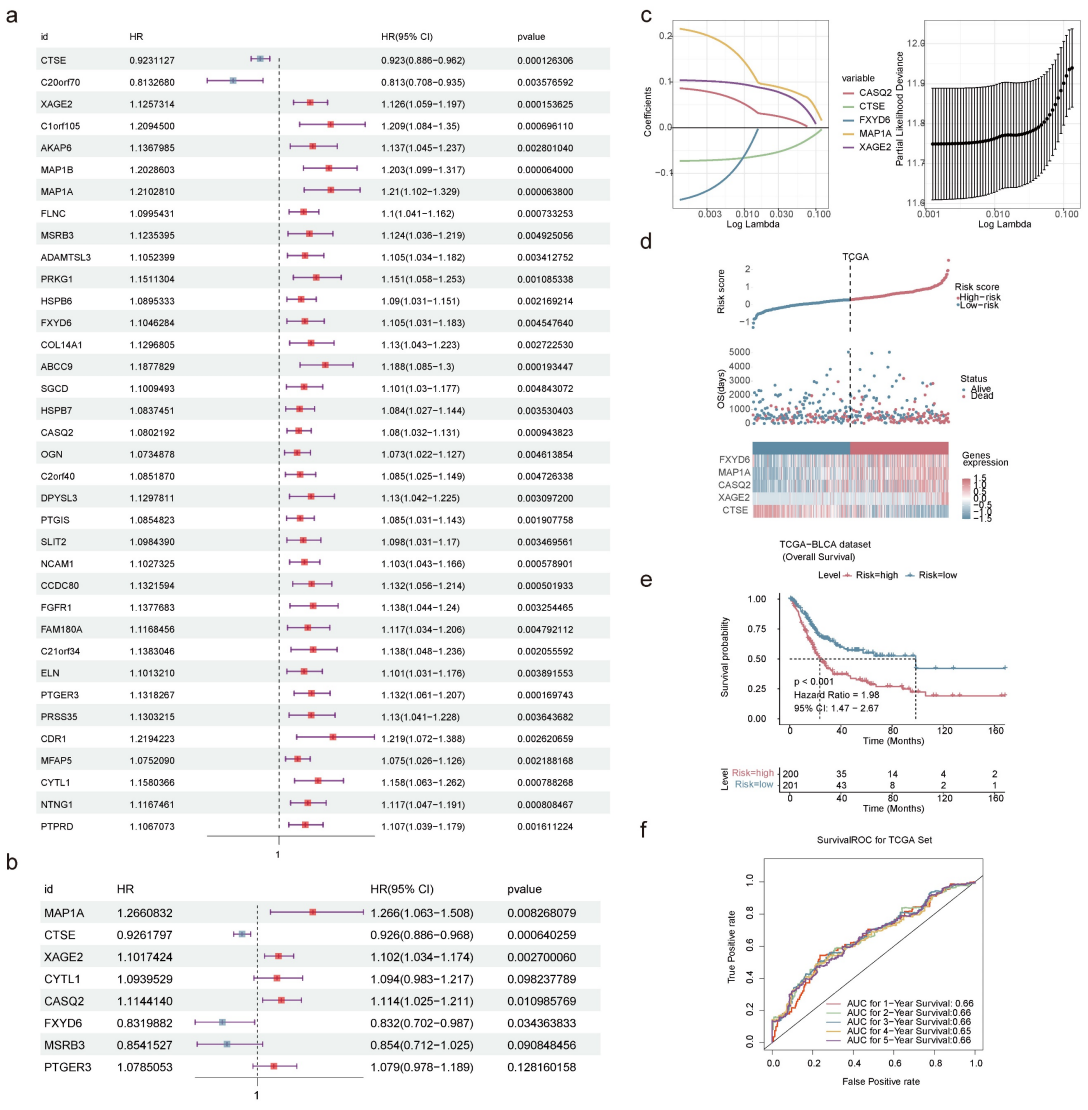
** Establishment and validation of prognostic models.** Forest plots of univariate **(a)** and multivariate **(b)** Cox regression analyses. The HR represents the risk ratio, lower 95% CI and upper 95% CI are 95% confidence intervals for risk values. **(c)** Screening of prognostic genes by LASSO regression analysis. Risk score **(d)** and K-M curve **(e)** predicting the overall survival and survival probability of high- and low-risk patients in TCGA-BLCA dataset. **(f)** Time-dependent ROC curves displaying the true positive rate of 1-5 years survival in TCGA-BLCA dataset.

The univariate Cox regression analysis produced 36 genes related to the survival of BLCA patients in the TCGA-BLCA dataset (**Figure 8a**). The PH hypothesis test evaluated the univariate Cox regression model. The outcomes indicated that the overall test p-value of the model was 0.67, and the linear relationship between the residual and time was not significant, suggesting that the model passed the PH test (Figure S11). Multivariate Cox regression analysis ascertained CTSE, XAGE2, MAP1A, CASQ2, and FXYD6 (**Figure 8b**). To decrease the false-positive rate and enhance the accuracy of the regression model, we developed a LASSO model to validate the prognostic significance of these five genes. Further LASSO model confirmed them as prognostic genes when the model error was minimal (**Figure 8c**). The equation for risk-score was: risk score=(-0.073) × CTSE + 0.104 × XAGE2 + 0.217 × MAP1A + 0.086 × CASQ2 + (-0.158) × FXYD6. The function annotations of the proteins encoded by five prognostic genes are listed in **Table 4**, with the majority of them participating in protein binding.

The proposed risk model, contrasting outcomes for BLCA patients, identified a reduced OS for those at higher risk (**Figure 8d**). K-M survival curves certified that low-risk BLCA patients had a longer lifespan (**Figure 8e**). Additionally, the AUC at 1-5 years exceeded 0.60, demonstrating the precise predictive performance of the risk model (**Figure 8f**). Furthermore, validation using the GSE13507 dataset was consistent with the TCGA-BLCA data in that there was a significant difference in survival between patients in the high- and low-risk groups; the 1-, 2-, 3-, and 5-year ROCs were greater than 0.6, which bolstering the model's evaluation potency (**Figure S12a-c**).

### 2.9 Nomogram model kept a high predictive precision

**Figure 9:**
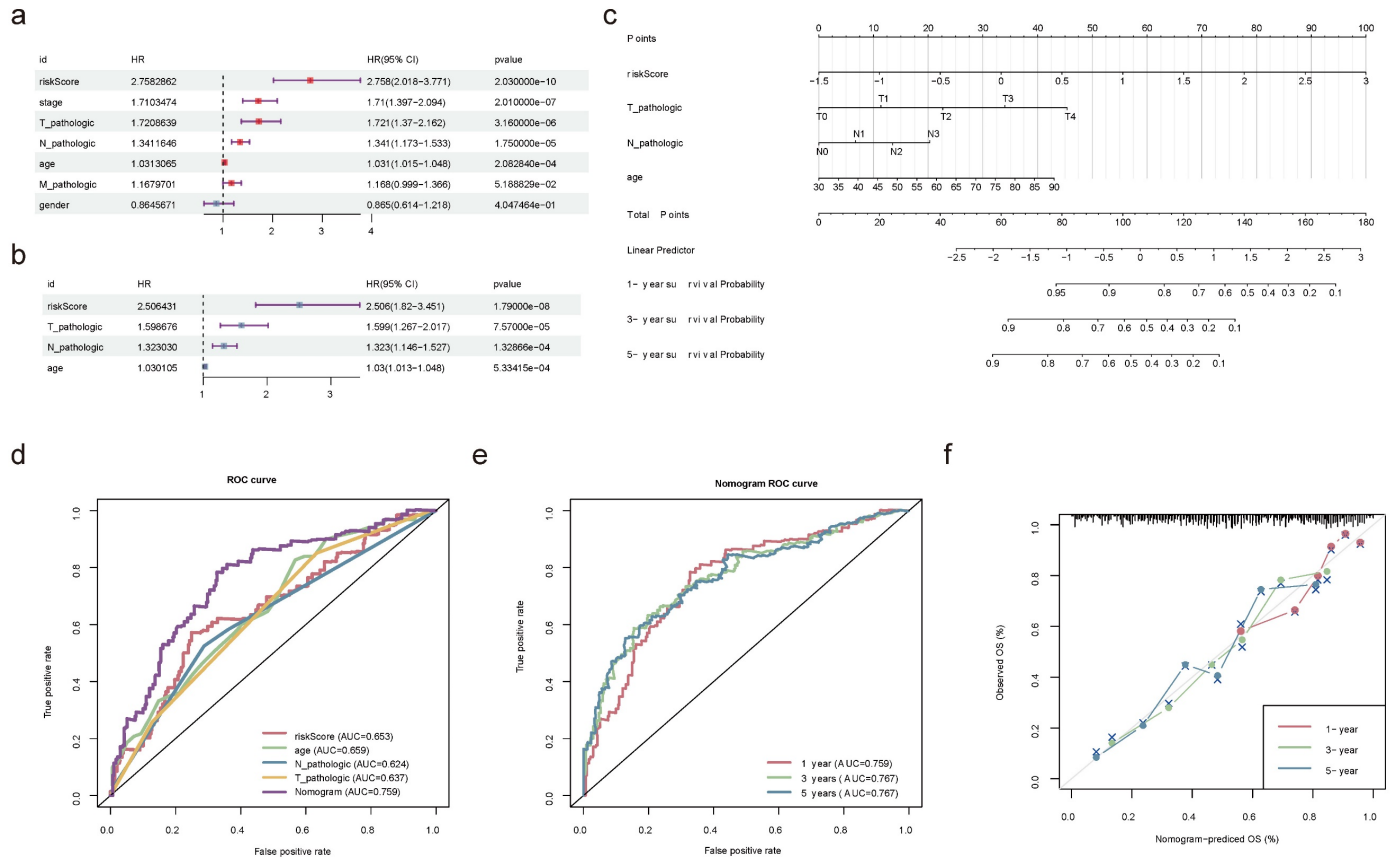
** Construction and validation of nomogram.** Forest plot summary of the univariate **(a)** and multivariable **(b)** Cox regression analyses of independent prognostic factors. **(c)** Nomogram model integrating the age, pathologic T, pathologic N, and risk score for predicting the survival probability of patients at 1-, 3- or 5-year. **(d)** ROC curves evaluating the predictive performance of the nomogram model. **(e)** ROC curves of the nomogram for predicting the survival outcomes at 1-, 3- or 5-year. **(f)** Calibration curves of the nomogram model for the overall survival of 1-year, 3-year and 5-year. The approximate 45-degree line represents the ideal prediction. (ns not significant, * P < 0.05, ** P < 0.01, *** P < 0.001).

Univariate and multivariate Cox regression analyses in the TCGA-BLCA dataset confirmed that age, pathologic T, pathologic N, and risk score were independent prognostic factors (**Figure 9a-b**). Based on these factors, a nomogram model was developed to demonstrate the survival probability of BLCA patients at 1-, 3-, and 5-year. Higher total points on the nomogram corresponded to lower survival probabilities for BLCA patients at these time points (**Figure 9c**). Ulteriorly, the accurately predictive performance of the nomogram model was illustrated by ROC curves and calibration curves. The true positive rate of the nomogram model exceeded that of any single factor (**Figure 9d-e**). Once again, the slope of the calibration curve was closed to 1, illustrating the high prediction accuracy of the nomogram model (**Figure 9f**). Summarily, the development of a nomogram model might be helpful for the prognosis assessment of BLCA patients.

### 2.10 Stratified survival analysis

**Figure 10:**
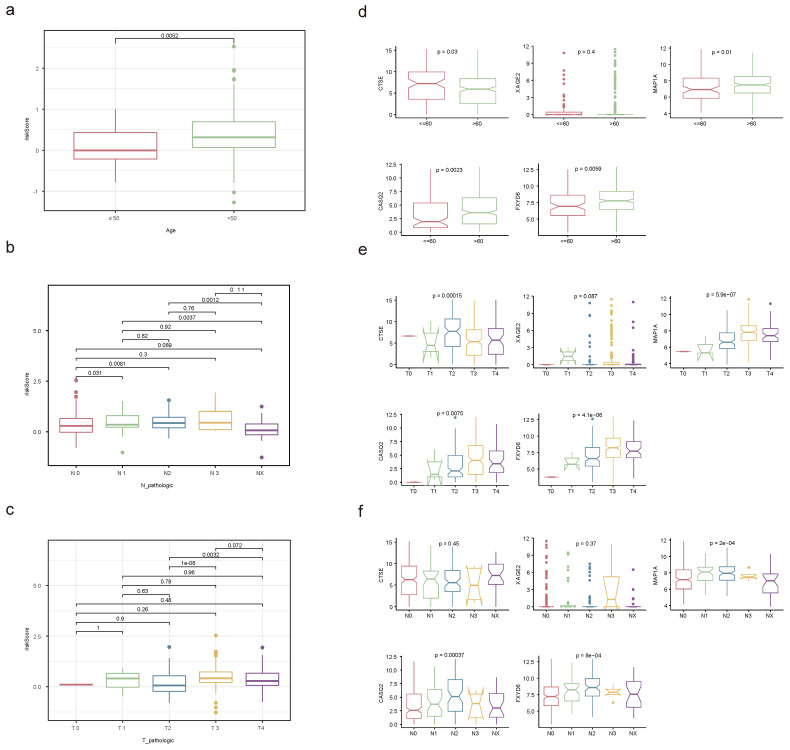
** Correlation analysis of clinicopathological factors with independent prognostic significance.** Discrepancies of risk score between age **(a)**, pathological N **(b)** and pathological T **(c)** subgroups through Wilcoxon rank-sum test. Expression levels of prognostic genes in age **(d)**, pathological T **(e)** and pathological N **(f)** subgroups. (ns not significant, * P < 0.05, ** P < 0.01, *** P < 0.001).

**Figure 11:**
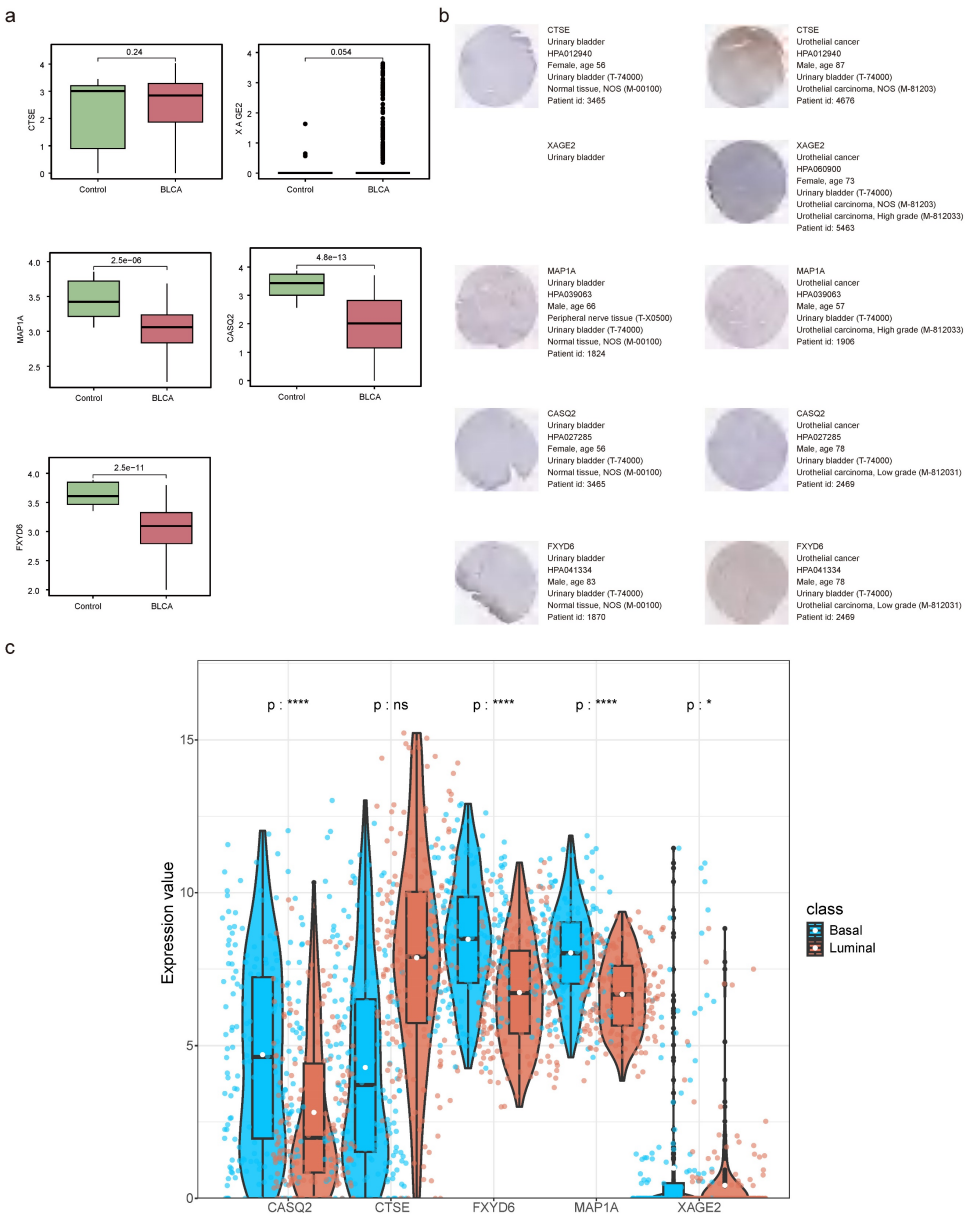
** Prognostic genes' expression detection. (a)** The expression levels of prognostic genes in BLCA and controls. **(b)** Staining landscape of prognostic genes in urinary bladder and urothelial cancer. (ns not significant, * P < 0.05, ** P < 0.01, *** P < 0.001). **(c)** Prognostic gene expression in molecular subtypes of bladder cancer.

The relationships between risk score and clinicopathological factors unveiled that risk scores elevated distinctly as the BLCA progressed from pathologic N0 to N2 stages, as were the risk scores from pathologic T2 to T3 stages (**Figure 10a-c**). Among the prognostic genes showing marked expression discrepancies between age, pathological T, and N subgroups, MAP1A, CASQ2, and FXYD6 exhibited high expression levels in patients over 60, whereas CTSE retained the opposite trend. Notably, the expression of CASQ2 and FXYD6 showed a gradual increase across pathological N0-N2 and T0-T3 stages (**Figure 10d-f, Figure S13**). Stratified survival analysis revealed no significant difference in survival between the T1+T2 subgroups in the two risk teams. However, the survival probabilities of high-risk patients in the other subgroups were memorably reduced (**Figure S14a-f**). Regarding the expression levels of prognostic genes between BLAC and controls in the TCGA-BLCA dataset, distinctly, MAP1A, CASQ2, and FXYD6 were lower expressed in BLCA tissues (**Figure 11a-b**). In addition, the prognostic genes XAGE2, MAP1A, CASQ2, and FXYD6 were significantly different in the basal and luminal subtypes. Among them, the expression of MAP1A, CASQ2, and FXYD6 was higher in the basal subtype, while XAGE2 was more highly expressed in the luminal subtype (**Figure 11c**).

### 2.11 Function and mutation analyses in two risk teams

**Figure 12:**
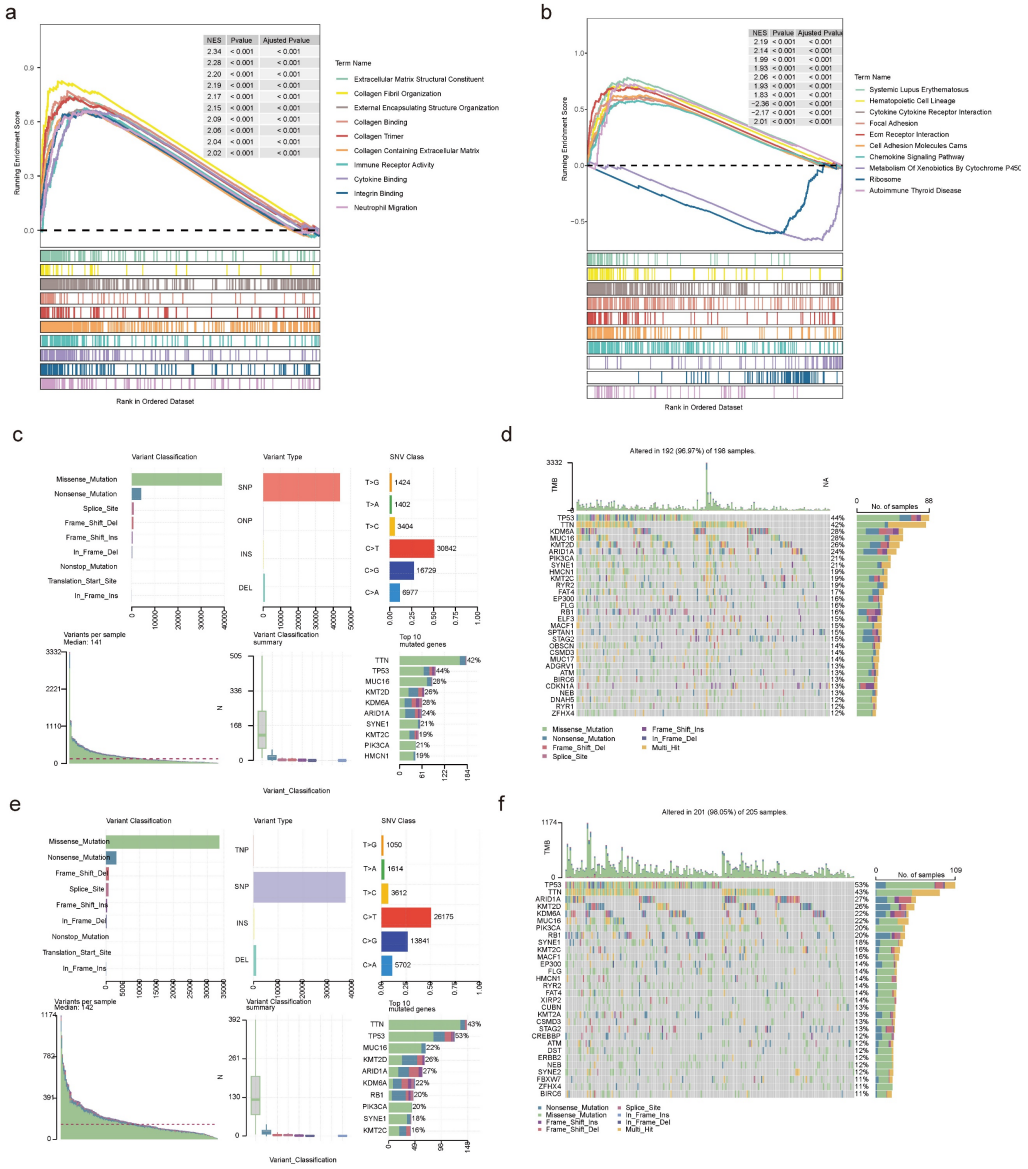
** Functional analysis and mutational landscape characterization between high- and low-risk teams.** GO **(a)** and KEGG **(b)** analyses between high- and low-risk teams utilizing GSEA method. Details of the 10 most frequently mutated genes in low-risk team** (c)** and high-risk team **(e)**. Waterfall plot illustrating the 10 mutated genes in low-risk team **(d)** and high-risk team** (f)**. The gene list is sorted by mutation frequency, with the corresponding mutation types color-coded on the right. The top section displays the TMB.

To investigate the enrichment pathways in the high- and low-risk teams, we observed 1,089 GO entries and 49 KEGG pathways involved by all genes in two risk teams, containing cytokine-cytokine receptor interactions, ECM receptor interactions, chemokine signaling pathways, ribosomes, autoimmune thyroid diseases, etc (**Figure 12a-b**). Further analysis of mutations in two risk teams revealed distinct patterns. In the low-risk team, the top 5 mutated genes were KMT2D, MUC16, KDM6A, TTN, and TP53, while the top 5 mutated genes in the high-risk team comprised KDM6A, KMT2D, ARID1A, TTN and TP53 (**Figure 12c-f**). Besides, there was no obvious discrepancy in TMB between the two risk teams (**Figure S15a**). Among the frequently mutated genes, TP53 and KDM6A exhibited mutations in both risk teams. The mutation rate of TP53 was higher in the high-risk team (86.29%) compared to the low-risk team (74.36%). Similarly, the mutation rate of KDM6A was higher in the low-risk team (47.00%) (**Figure S15b-c**).

### 2.12 Immune cell infiltration in BLCA

**Figure 13:**
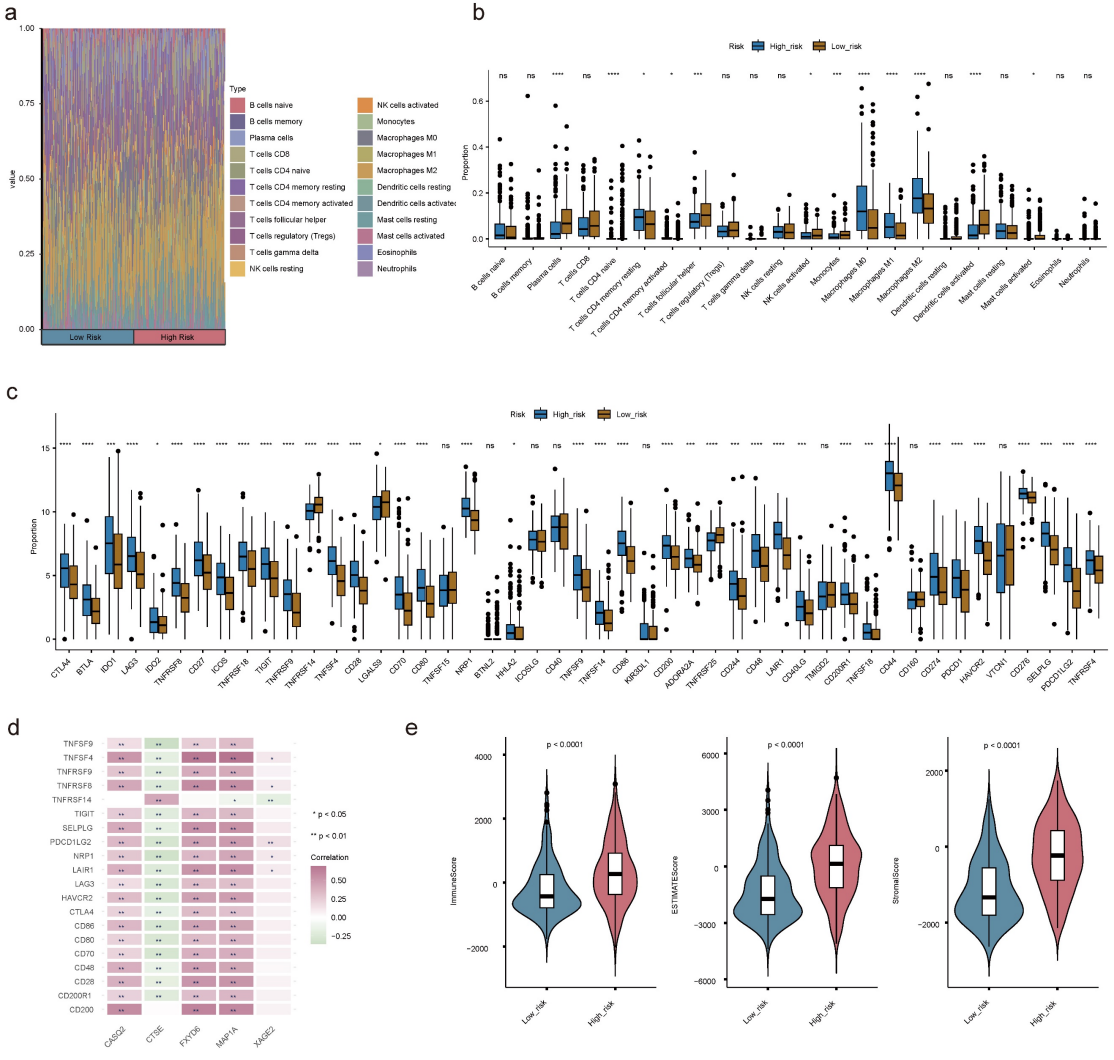
** Immune cell infiltration (ICI) analyses. (a)** Heat map demonstrating the proportion of 22 immune cells in high- and low-risk teams. **(b)** Box plot showing the different proportions of 22 immune cells. **(c)** The expression levels of immune checkpoint-related genes in the two risk teams. **(d)** Correlation matrix of prognostic genes and discrepant immune checkpoint-related genes. **(e)** The discrepancies of immune score, ESTIMATE score and stromal score between two risk teams. (ns not significant, * P < 0.05, ** P < 0.01, *** P < 0.001).

Understanding the immune cell infiltration in BLCA was crucial for evaluating the prognosis of BLCA patients. Therefore, we analyzed to detect the infiltration of immune cells in high and low risk groups. Among the 22 types of immune cells, the proportion of macrophages was relatively high (**Figure 13a**). There were 12 discrepant immune cells (plasma cells, naive CD4^+^ T cells, activated/resting memory CD4^+^ T cells, T follicular helper (Tfh) cells, activated natural killer (NK) cells, monocytes, M0 Macrophages, M1 Macrophages, M2 Macrophages, activated mast cells and activated dendritic cells (DCs)). We found the high-risk team had a lower proportion of plasma cells, activated DCs, Monocytes, activated NK cells, activated mast cells, and Tfh cells (**Figure 13b**). Moreover, there was an obvious correlation between discrepant immune cells and prognostic genes, for instance, M0 Macrophages were positively correlated with other prognostic genes except CTSE (**Figure S16a-e**). The expression levels of immune checkpoints were observably updiscrepant between two risk teams, most of them were elevated in the high-risk team, such as CTLA4, LAG3, and so forth (**Figure 13c**). Spearman's correlation analysis between prognostic genes and discrepant immune checkpoints underscored that CTSE displayed negative correlations with the most discrepant immune checkpoints in addition to CD200 and TNFRSF14 (**Figure 13d**). The immune score, stromal score, and ESTIMATE score of the high-risk team were observably higher than those of the low-risk team (**Figure 13e**).

### 2.13 Drug sensitivity analysis

**Figure 14:**
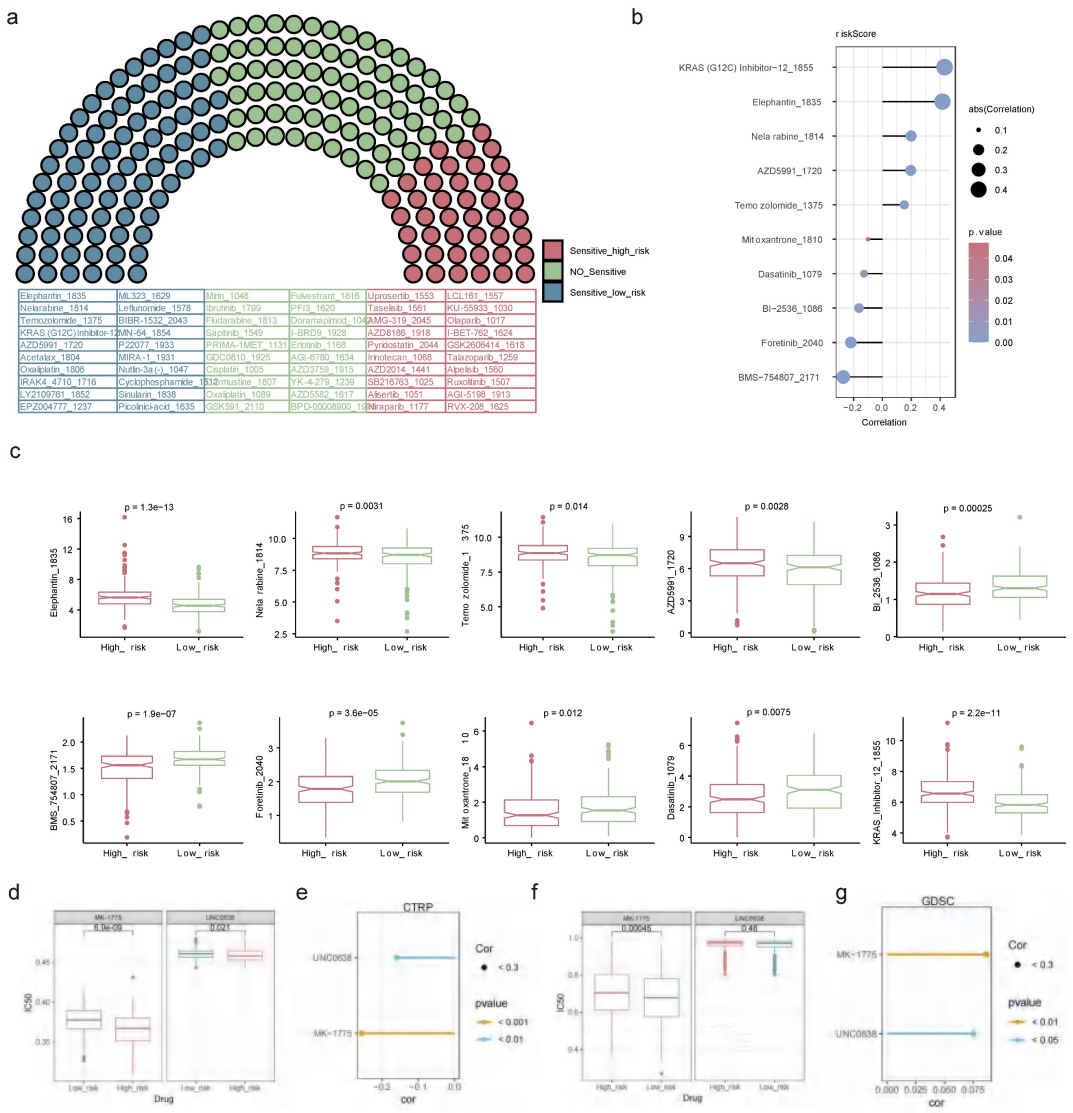
** Drug sensitivity analysis. (a)** Assessment of drug sensitivity of high- and low-risk patients to 198 kinds of drugs. **(b)** The relationships of top 5 sensitive drugs in the high/low-risk teams and risk score. **(c)** Discrepancies in sensitivity of high- and low-risk patients to top 5 sensitive drugs in the high/low-risk teams. Obvious correlations between UNC0638, MK-1775 and risk score in CTPR **(e)** and GDSC **(g)** databases. IC50 differences between UNC0638 and MK-1775 in CTPR **(d)** and GDSC **(f)** databases. (ns not significant, * P < 0.05, ** P < 0.01, *** P < 0.001).

Out of the 198 drugs, the high-risk patients exhibited greater sensitivity to 43 drugs such as Uprosertib 1553, AMG-319 2045, Pyridostatin 2044, and other drugs, while the low-risk patients showed higher sensitivity to 81 drugs embracing Temozolomide 1375, AZD5991 1720 and so on (**Figure 14a**). The relationships of the top 5 sensitive drugs in the high/low-risk teams and risk score elucidated that KRAS (G12C) Inhibitor-12 1855 and BMS-754807 2171 showed the strongest positive and negative associations with risk scores, respectively (**Figure 14b**). Additionally, obvious discrepancies were observed between the two risk patients for these 10 drugs. Detailedly, the high-risk patients were less sensitive to Elephantin 1835, Nelarabine 1814, Temozolomide 1375, AZD5991 1720, and KRAS 12 1855, while were highly sensitive to Bl 2536 1086, BMS 754807 2171, Foretinib 2040, Mitoxantrone 1810 and Dasatinib 1079 (**Figure 14c**). A lower IC50 value indicated that the targets were more sensitive to the inhibitory effects of the drugs, suggesting that the patient might be more responsive to treatment. This meaned that the drug was more effective in treating the disease, so it could be hypothesised that BLCA patients were more likely to have a positive therapeutic response to drugs such as Bl 2536 1086, BMS 754807 2171, Foretinib 2040, Mitoxantrone 1810 and Dasatinib 1079. In the CTRP dataset, high-risk patients were more sensitive to both drugs significantly (**Figure 14d**), and these drugs showed a negative correlation with the risk score (**Figure 14e**). MK-1775, a WEE1 inhibitor, showed potential value in the treatment of BLCA. Similarly, UNC0638 as a specific inhibitor against G9a, showed potential value in the treatment of BLCA. In the GDSC dataset, low-risk patients demonstrated higher sensitivity to MK-1775, whereas high- and low-risk patients did not exhibit a significant difference in sensitivity to UNC0638 (**Figure 14f**). Furthermore, both compounds showed a positive correlation with the risk scores (**Figure 14g**).

### 2.14 The workflow of this study

**Figure 15:**
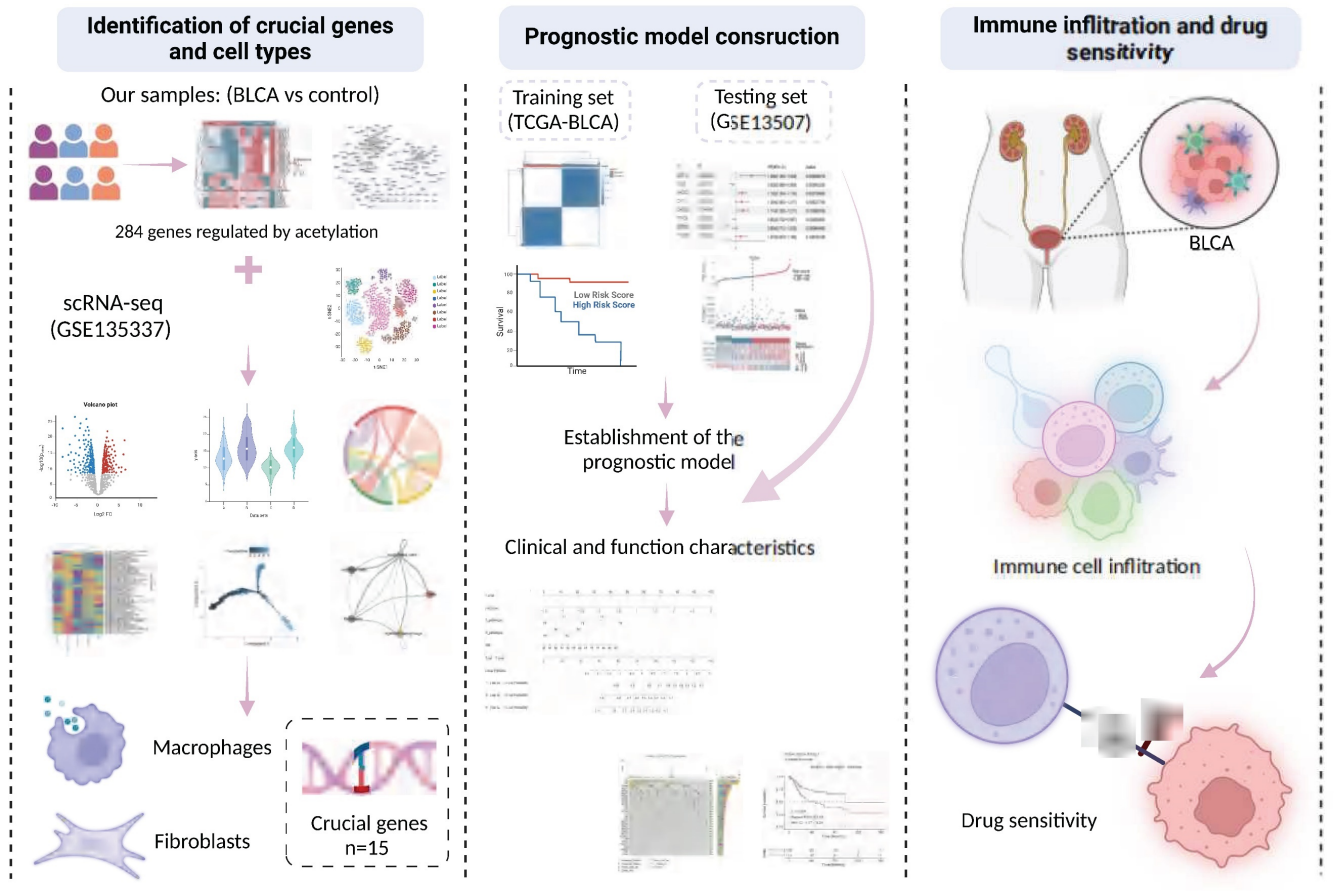
Graphic abstract of this study.

The tumor tissue samples (n=6) were collected from patients diagnosed with BLCA, along with paraneoplastic tissue samples (n=6) as controls. These were subjected to comprehensive profiling, including transcriptome sequencing, proteomic sequencing, and protein acetylation profiling. Additionally, we conducted a comprehensive reanalysis of multiple previously published cohorts to train and validate our predictive model. These analyses included two bulk RNA cohorts (TCGA-BLCA, and GSE13507) as well as one scRNA dataset (GSE135337). A flowchart illustrating the study design is presented in **Figure 15**.

## 3. Discussion

BLCA is a prevalent malignancy of the urological system, which is the tenth most common reason for cancer and the thirteenth most common cause of cancer deaths globally, with gender-specific differences in incidence and prognosis, mainly affecting the > 55-year-old and male populations^11^. Tumor recurrence after complete resection and advanced tumors continue to be a formidable challenge in the treatment of BLCA, and thus to address this issue, several studies have elucidated the heterogeneous molecular landscapes used for the typing and treatment of BLCA from a multi-omics perspective^12^,^13^. Understanding the relationships between DEGs, DEPs, and DEPAs is fundamental to unraveling the complex regulatory networks in BLCA. DEGs represent changes at the transcriptional level, while DEPs reflect these changes at the protein expression level. Further, DEPAs provide insights into how these proteins are modified post-translationally through acetylation, impacting their function and stability. By integrating data from these three molecular layers, we can uncover how alterations in gene expression lead to changes in protein abundance and how these proteins' acetylation states further influence their roles in tumor progression. For example, modifications in post-translational histone acetylation in various tumor entities, including BLCA, have been identified, and the investigation of their tumor microenvironment and pathway alterations could facilitate our understanding of the critical molecules driving tumors^9^,^14^. The purpose of the present study was to investigate the roles of genes regulated by protein acetylation in patients with BLCA and to reveal their value as prognostic genes, ultimately aiming to inform individualized treatment strategies, including the selection of appropriate targeted agents and personalized treatment regimens, to improve treatment efficacy and reduce adverse effects.

Five acetylation-related genes were identified in our study, among which CTSE is an intracellular aspartic, encoding an A1-family peptidase that plays an immune role in the tumor microenvironment by regulating antigen presentation and chemotaxis^15^. The role of CTSE in BLCA is paradoxical, as it was found in a study by Wild, *et al.*^16^ that CTSE is predominantly overexpressed in NMIBC (pTa, pT1, and pT2), and its expression in pTa stage tumors correlates with tumor progression characteristics, but overall OS was significantly longer in BLCA patients with strong CTSE immunohistochemistry positivity than in the CTSE-negative group (178 vs. 140 months, p=0.003), which is similar to the results of the present study. CTSE expression was highest at the T2 stage, yet served as a protective factor for the prognosis of BLCA patients in the risk model. However opposite results were obtained in a recent study, which showed that CTSE was highly expressed in BLCA tissues relative to normal tissues, and silencing of CTSE suppressed proliferation, migration, and invasion of the 5637 cell line^17^. The expression of CTSE in the present study was not significantly different between BLCA and controls, which may be related to the fact that a greater proportion of patients in the TCGA dataset with T3- 4 stage, so more experiments are needed to verify the effect of CTSE in BLCA tumorigenesis and progression. MAP1A has been suggested to be strongly associated with patient prognosis, immune infiltration, and autophagy in several BLCA prediction models, and was validated to exhibit decreased expression levels in BLCA tissues and several BLCA cell lines^18^,^19^, which is consistent with the results of the current study. XAGE2, which has not been reported in the literature in BLCA, is considered a cancer/testis-associated gene, and is frequently found to be aberrantly expressed in melanoma and Ewing sarcoma^20^, therefore it was the first time revealed in this study to be associated with the prognosis of BLCA patients. FXYD6 and CASQ2, which are involved in ion channel activity, have only been mentioned in a small number of bioinformatics analyses of BLCA without the support of real-world data^21^,^22^, but have been revealed to promote tumor progression and regulate tumor-microenvironmental interactions in hepatocellular carcinoma and breast cancer^23^,^24^. CTSE and MAP1A were found to be associated with patient prognosis as in previous studies. While XAGE2, FXYD6 and CASQ2 are newly identified prognostic genes in this study, which provide new therapeutic targets in this field.

Additionally, recent studies have identified new therapeutic targets in BLCA, further broadening the scope of potential interventions. For instance, studies have highlighted the significance of RNA methylation regulators like METTL3 and ALKBH5 in shaping tumor behavior by modulating m6A RNA modifications, providing promising avenues for BLCA treatment^25^,^26^. Another study revealed that SDC1, a key regulator of epithelial-mesenchymal transition (EMT), plays a pivotal role in driving invasion and metastasis in BLCA^27^. These findings underscore the critical need for comprehensive investigations into emerging molecular targets, as they hold great potential for improving therapeutic outcomes in BLCA patients.

The risk model established based on the above five critical genes—CTSE, MAP1A, FXYD6, XAGE2, and CASQ2—classifies BLCA patients into high- and low-risk groups, revealing significant differences in various aspects of clinicopathologic features, prognosis, and treatment outcomes. This classification allows for a more nuanced understanding of patient stratification and helps tailor treatment approaches. However, the predictive efficacy of this model in overall survival (OS) was not entirely satisfactory, with the 1, 3, and 5-year ROC curves yielding values around 0.66, which is below our desired threshold for clinical applicability. Recognizing that the prognosis of BLCA is influenced by a multitude of factors beyond genetic expression alone, several studies have underscored the importance of incorporating clinicopathologic characteristics into prognostic models to enhance predictive accuracy^15^,^19^,^28^. Building on this insight, we developed an enhanced prognostic model that integrates age, pathological T/N stage, and risk score derived from the initial five-gene model. This integrative approach acknowledges the complex interplay between genetic and clinical factors in determining patient outcomes. The resulting model demonstrated a slope of the calibration curve close to 1, indicating a high level of agreement between predicted and observed outcomes. Consequently, the predictive efficacy for 1, 3, and 5-year OS was significantly improved, with ROC values ranging from 0.759 to 0.767. This marked improvement underscores the robustness of our enhanced model, providing a more reliable and accurate tool for predicting the prognosis of BLCA patients. The integration of clinicopathologic characteristics not only refines the risk stratification but also supports personalized treatment regimens, ultimately aiming to improve patient management and clinical outcomes in BLCA.

Previous studies have shown that histone acetylation plays an important role in regulating the tumor microenvironment (TME), and histone deacetylase (HDAC) inhibitor CM-1758 can increase CD8^+^ T cell infiltration and promote macrophage polarization toward M1-like in the TME of BLCA patients, representing an attractive target for immunotherapy^29^. The results of GSEA analysis in our study also suggested significant differences in inflammation and immune-relevant pathways across the high and low-risk groups, so we further conducted a detailed analysis of immune cell infiltration in both groups and observed that a variety of immune cells including macrophages, activated NK cells, monocytes, and activated DCs were significantly divergent infiltration. Macrophages represent one of the major compartments in the TME, and earlier studies have demonstrated that signal transducer and activator of transcription 6 (STAT6) was acetylated by acetyltransferase CREB-binding protein (CBP), which inhibits macrophage M2 polarization resulting in the enhancement of anti-tumor immunity in macrophages^30^. Since one of the crucial cell types identified in the present study contained macrophages as well, and all of the five prognostic genes, except CTSE, were positively correlated with macrophage infiltration, it was suggested that modulation of macrophages in the TME by histone acetylation may be beneficial in enhancing anti-tumor immunity in BLCA patients. To comprehensively evaluate the role of the risk model in TME of BLCA patients, we further analyzed the immune checkpoints and immune scores of both groups, which revealed that the expression levels of most immune checkpoints were elevated in the high-risk group with higher immune scores, stromal scores, and ESTIMATE scores, suggesting that there may be differences in the immune properties across the two groups, where diverse elements in the complex TME interact to shape tumor behavior and its response to treatment.

Finally, we additionally characterized the sensitivity towards potential drug treatments in the two groups of BLCA patients. Among 198 drugs, patients in the high-risk group were more sensitive to drugs such as pan-Akt pathway inhibitor (uprosertib) and a selective PI3Kδ inhibitor (AMG-319), as a critical pathway and molecule modulating the immune response *in vitro* and *in vivo*, which can inhibit cytokine regulation, PD-L1 expression, and tumor-infiltrating Regulatory T cells (Treg), and currently used in the treatment of breast cancer, colon cancer and B cell malignancies^31^-^33^. In contrast, low-risk patients show higher sensitivity to drugs like Temozolomide and selective MCL-1 inhibitor (AZD5991). Temozolomide penetrates the blood-tumor barrier efficiently and causes cytotoxicity by inducing DNA double-strand breaks, representing a first-line chemotherapeutic agent for glioblastoma^34^. AZD5991 binds directly to Mcl-1 and induces rapid apoptosis in tumor cells through activation of the Bak-dependent mitochondrial apoptotic pathway, which is currently undergoing clinical trials in patients with hematologic malignancies^35^. It is believed that more patients could benefit from targeted therapies in the future through precise risk stratification.

To summarize, following the identification and validation of five histone acetylation-related prognostic genes and two crucial cell types from the perspectives of multi-omics and multiple datasets, and the establishment of a risk model that can accurately predict the prognosis of the patients in our study, the BLCA patients were divided into high- and low-risk groups, with significant differences in the prognosis, clinicopathological characteristics, immune microenvironment, and drug susceptibility of the patients in the two groups, proposing promising targets for the stratified diagnosis and treatment of BLCA. However, there are still some undeniable limitations in this study. Firstly, the sample size used for testing was relatively small, with only 6 samples, which may affect the robustness and generalizability of our findings. To address this, future studies will include a larger cohort of BLCA patients to validate and refine the risk model. Secondly, the expression of prognostic genes in BLCA tissues and cells has not been validated in the real world, leading to potential deviations from the analysis of data from public databases. We plan to conduct extensive validation using real-world samples from diverse clinical settings to ensure the reliability of our findings. Lastly, more cellular or molecular-level experiments are required to verify the detailed mechanisms by which prognostic genes affect the prognosis of BLCA. Future research will focus on performing these experiments to elucidate the biological pathways involved, thereby strengthening the mechanistic understanding and clinical applicability of the identified prognostic genes. Addressing these limitations in future studies will be crucial for confirming our findings and enhancing the clinical applicability of our risk model.

## 4. Materials and Methods

### 4.1 Data collection and processing

The tumor tissue samples (n = 6) were collected from patients diagnosed with BLCA, along with paraneoplastic tissue samples (n = 6) as controls. These were subjected to comprehensive profiling, including transcriptome sequencing, proteomic sequencing, and protein acetylation profiling. To mitigate potential disparities between the BLCA specimens and controls and to condense the multidimensional data, we employed Principal Component Analysis (PCA) on both the transcriptomic and protein acetylation datasets. We utilized the MaxQuant-Andromeda software to filter the low-quality protein data (FDR < 0.05, default parameter) and PCA to examine inter-group heterogeneity and reproducibility of samples within the BLCA and control groups.

The gene expression profiles, clinical information, prognostic information, tumor mutational burden (TMB) data, and copy number variations (CNV) data of the TCGA-BLCA dataset were obtained from The Cancer Genome Atlas (TCGA, https://www.cancer.gov/ccg/research/genome-sequencing/tcga) database. The TCGA-BLCA dataset encompassed 402 BLCA tumor tissue samples and 19 normal tissue samples. For the pre-processing method of the TCGA-BLCA dataset, we used the GDC mRNA quantitative analysis process to measure gene level expression. The specific process involves processing of raw read counts using STAR. For more details, please refer to https://docs.gdc.cancer.gov/Data/Bioinformatics_Pipelines/Expression_mRNA_Pipeline/. The single-cell RNA sequencing (scRNA-seq) dataset GSE135337 of BLCA, and the transcriptome dataset GSE13507 (survival information), were acquired from the Gene Expression Omnibus (GEO) database (https://www.ncbi.nlm.nih.gov/geo/). The GSE135337 comprised 7 BLCA tumor tissue samples and 1 paraneoplastic tissue sample, while GSE13507 included a total of 165 BLCA tumor tissue samples after filtering out samples with incomplete clinical data^36^,^37^. The GSE13507 dataset was preprocessed as follows: array data were processed using Illumina BeadStudio software. We used Quantile normalisation and log2 transformation to process the protein gene expression data. To export the data matrix, the "Sample Gene Profile" option was selected in the software. For RNA-seq reads, STAR (version 2.0.4) was used to compare with the Ensembl 76 top component. For RNA-seq gene counts, Subread:featureCount (v1.4.5) and htseq (v0.8.0) were used for count derivation, and RSeQC (v2.3) was used for quality control of RNA-seq data. While gene RPKM quantification and normalisation was performed by Partek Genomics Suite software version 6.6.

In this study, we firstly performed a multi-omics joint analysis to screen and obtain 284 candidate genes, and then took the intersection with the differential genes in the single-cell dataset to finally obtain 15 key genes. Then, based on these 15 key genes, TCGA-BLCA patients were consistently clustered to obtain two subtypes. Subsequently, the two subtypes as well as the BLCA/Control samples were analysed differentially in the TCGA-BLCA dataset respectively, and finally 318 candidate prognostic genes were obtained. Five prognostic genes were obtained by single-factor cox, multifactor cox and lsaao regression screening, and their risk scores were calculated to establish risk models. At the same time, the reliability of the risk model was verified in the TCGA-BLCA dataset and the GSE13507 dataset. Thus, the joint analysis of transcriptome, proteome, acetylated proteome, single cell dataset, TCGA-BLCA dataset and GSE13507 dataset was achieved.

### 4.2 Differential expression analysis

To identify genes with significant differences in gene expression between the different sample groups in the self-sequencing transcriptome dataset (differential expression genes, DEGs), the R package DESeq2 (version 1.36.0) was used for the analysis (|log2FC| > 0.5, p-value < 0.05). Subsequently, differences and correlations between BLCA and control group proteins in the self-sequencing proteomic dataset were investigated using MaxQuant-Andromeda software (FDR < 0.05, thresholds could be adjusted according to screening) (differential expression proteins, DEPs). Finally, to identify acetylated proteins that differed between the BLCA and control groups in the self-sequencing proteomic acetylation dataset (differential expression acetylated proteins, DEPAs), the threshold was set at BLCA/Control > 1.2 (or BLCA/Control < 0.8) with a P value < 0.05^38^. Subsequently, the R package 'clusterProfiler' (v 4.6.2)^39^ was utilized for Gene Ontology (GO) and Kyoto Encyclopedia of Genes (KEGG) enrichment analyses to investigate latent functions of DEGs, DEPs, and DEPAs (p-value < 0.05 and count ≥ 1). Additionally, subcellular localization analysis was implemented utilizing the WoLF PSORT software (https://wolfpsort.hgc.jp/) to predict the position of DEPAs in subcellular fraction.

### 4.3 Screening and functional annotation of candidate genes regulated by acetylation

In order to understand the expression between differential genes and differential proteins, the intersection of differential genes/differential proteins with the same expression trend was taken separately and then the result was taken as a concatenation to get intersection genes/intersection proteins. Subsequently, to obtain the candidate genes (PA-DEGs), we performed spearman correlation analysis (|R| > 0.6 and p-value < 0.05) of intersection genes/intersection proteins with DEPAs. Functional annotation was supported by the R package 'clusterProfiler' to explore the biological functions served by each candidate gene (p-value < 0.05 and count ≥ 1). Additionally, to understand the relationships among candidate genes at the protein level, a protein-protein interaction (PPI) network was synthesized through the Search Tool for the Retrieval of Interacting Genes (STRING, https://www.string-db.org/) database and visualized via Cytoscape software (v 3.7.2) (Confidence coefficient > 0.9)^39^.

### 4.4 Quality control of scRNA-seq data

Quality control was performed on the single-cell dataset GSE135337 using the R package 'Seurat' (v 4.3.0.1)^40^. Parameters min. cells = 3 and min. features = 200 were set to create 'Seurat' objects, thereby filtering out the large number of pseudocells generated during the sequencing process. The double cell rate was set at 8% for samples with more than 10,000 cells and 5% for samples with less than 10,000 cells. The library size was required to be greater than 500 and less than 95% when the number of cells was less than 10,000, and greater than 500 and less than 92% when the number of cells was greater than 10,000. Additionally, mitochondrial content was restricted to less than 10%. Subsequently, 2,000 genes with the highest standardized variance were identified using the 'vst' method in the 'FindVariableFeatures' function after data standardization. Integration of the data was performed using the 'FindIntegrationAnchors' and 'IntegrateData' functions to mitigate batch effects. Linear transformations were applied for scaling, followed by PCA to standardize gene variance and maintain equal weight. Regression correction based on library size and gene number was applied to minimize bias from highly expressed genes. Furthermore, normalization ensured uniform cell distribution and outlier removal. The standard deviation of principal components was calculated using the 'ScoreJackStraw' function for further analysis.

### 4.5 Cell annotation

The 'FinBLCAeighbors' and 'FindClusters' functions were utilized for unsupervised clustering analysis. The 'FindClusters' was opted for obtaining cell clusters via grouping cells iteratively with the resolution being 0.3. Cell clusters were visualized by uniform manifold approximation and projection (UMAP). Furthermore, marker genes of cell clusters were identified via 'FindAllMarkers' and compared with marker genes of each cell type in the CellMarker database (http://biocc.hrbmu.edu.cn/CellMarker/ or http://bio-bigdata.hrbmu.edu.cn/CellMarker/) to obtain cell types. Besides, the proportions of cell types in every sample of the scRNA dataset were illustrated in bar graphs. The discrepancies in proportions of each cell type between BLCA and controls were also depicted.

### 4.6 Identification of key genes

DEGs in different cell types of BLCA and normal control were obtained using the "FindMarkers" function (log2FC > 0.5, p-value < 0.05, Minpct = 0.25) and these DEGs were taken in concatenated sets to obtain scRNA-DEGs. The intersection of scRNA-DEGs and PA-DEGs was identified as key genes. The 'circlize' package (v 0.4.15)^9^ was employed to reveal relationships among key genes. Ulteriorly, other genes maintaining similar functions with key genes were predicted in the GeneMANIA database (https://genemania.org/), and a gene-gene interaction (GGI) network revealing the interactions and functions among genes was developed.

### 4.7 Screening of crucial cell types

To clarify the differences in expression levels of key genes in BLCA samples and control samples across cell types in the single-cell dataset GSE135337, we performed estimations. The cell types in which key genes expressed variously were selected as crucial cell types (p-value < 0.05). To explore variations of pathways in which cell types were enriched, we performed gene set variation analysis (GSVA). Subsequently, heterogeneity analysis was performed on crucial cell types to reveal the diversity and characteristics within these specific cell types. Concretely, the data of crucial cell types were extracted, followed by secondary dimensionality reduction and clustering to annotate crucial cell types into distinct subclusters. Subclusters were displayed using UMAP, and the discrepant proportion of subclusters between BLCA and controls in crucial cell types was displayed by a bar chart.

### 4.8 Pseudo-temporal trajectory and cell communication analyses of crucial cell types

To identify differentiation differences of crucial cell types, the differential genes between BLCA and controls in crucial cell types were accessed via the 'differentialGeneTest' functions, pseudo-temporal trajectory analysis of crucial cell types was then accomplished through the R package 'Monocle' (v 2.30.0)^10^. Moreover, the expression of key genes in different branches was defined to understand the changes in pivotal gene expression in crucial cell types at different time points. To understand the ways cell types communicated, cell communication analysis proceeded by analyzing the ligand-receptor relationship of the features in the single-cell expression profile in the CellPhoneDB database.

### 4.9 Unsupervised consensus clustering

In order to better identify BLCA-related subtypes, clustering analysis was conducted on 402 BLCA tumor tissue samples from the TCGA-BLCA dataset using the R package 'ConsensusClusterPlus' (v 1.60.0)^41^. To ensure robust classification, the clustering process was iterated 100 times, with the optimal 'k' value (number of clusters) determined based on the relative change in the area under the cumulative distribution function (CDF) curves and the consensus matrix. Subsequently, Kaplan-Meier (K-M) survival curves were generated using the R package 'survminer' (v 0.4.9)^42^ to assess survival differences among different clusters (p-value < 0.05). Differential expression analysis was performed using the R package 'DESeq2' to identify Cluster-DEGs between clusters and BLCA-DEGs between BLCA samples and controls in the TCGA-BLCA dataset (|Log_2_FC| > 2 and adj. p-value < 0.05). The functions of Cluster-DEGs were explored through GO and KEGG enrichment analysis (adj. p-value < 0.05 and count ≥ 1). Candidate prognostic genes were identified by intersecting Cluster-DEGs with BLCA-DEGs.

### 4.10 Construction of a risk model

In TCGA-BLCA dataset, we used univariate Cox regression analysis (p-value < 0.005), Proportional Hazards (PH) assumption test (p-value > 0.05), multivariate Cox regression analysis (p-value < 0.05), and the least absolute shrinkage and selection operator (LASSO) regression algorithm, employed using 'survival' (v 3.5-7) and 'glmnet' (v 4.1-8) packages in R, to compute the weight for each variable^43^,^44^. In this case, PH assumes the underlying assumption that the effect of covariates on survival does not change over time, i.e., the risk ratio h(t)/h0(t) is fixed. We tested the Cox proportional risk model using the Schoenfeld residual method, which requires the residuals to be independent of time. If the results show a non-random relationship between the residuals and time, it indicates a violation of the proportional risk assumption. Therefore, the p-value for the PH test needs to be greater than 0.05. In contrast, the Lasso regression uses 10-fold cross-validation, where the sample data are randomly divided into 10 subsamples. Nine of these subsamples are used to estimate the model, and the resulting model was used to predict the explanatory variables for the remaining 1 subsample to obtain the mean square error (MSE). This was repeated to obtain 10 mean square errors, the average of which was the cross-validated MSE for the entire sample data. Finally, The prognostic genes were identified for use in constructing the risk prediction model with the following formula: 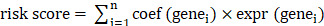. Moreover, the BLCA patients in TCGA-BLCA dataset (the number of cases in the high and low risk groups were 201 and 200 respectively) were categorized as high- and low- risk teams in compliance with median risk score. The R packages 'ggplot2' (v 3.3.5) and 'survminer' (v 0.4.9) were exploited to create a risk curve and K-M survival curve to assess the prognostic ability of risk model. In addition, the predictive performance of the risk model was evaluated by the receiver operating characteristic (ROC) curves, which were plotted using the R package 'survivalROC' (v 1.0.3.1). In the GSE13507 validation set (the number of cases in the high and low risk groups were 83 and 82 respectively), risk curves and prognostic gene heatmaps were plotted using the R package ggplot2, respectively. Meanwhile, KM curves and ROC curves of high and low risk groups were plotted using R package survminer (version 0.4.9) and R package survivalROC (version 1.0.3) as a way to validate the predictive effect of the risk model.

### 4.11 Independent prognostic analysis

To determine whether the risk model possessed independent prognostic value, and to thoroughly investigate other independent prognostic factors predicting the prognosis of BLCA patients, gender, age, stage, risk score, as well as pathologic T, N, and M stages were included in univariate Cox regression analysis (p-value < 0.05), Proportional Hazards (PH) hypothesis test (p-value > 0.05), and multivariate Cox regression analysis (p-value < 0.05) in the samples of the TCGA-BLCA dataset to identify independent prognostic factors. Subsequently, a nomogram model was developed to predict patient prognosis incorporating these factors. Importantly, ROC and calibration curves were created to assess the clinical application value of the nomogram model.

### 4.12 Stratified survival analysis

To explore the relationships between various clinicopathological factors with independent prognostic significance and risk score, as well as between clinicopathological factors and prognostic genes, and clinicopathological factors and overall survival (OS). The discrepancies in risk score, prognostic genes' expression, and high/low-risk patients' survival between clinical subgroups were estimated by the Wilcoxon rank-sum test in the TCGA-BLCA data (N = 401) (p-value < 0.05).

### 4.13 The expression of prognostic genes

To understand the expression patterns of prognostic genes in the TCGA-BLCA dataset, the expression levels between BLAC and controls were demonstrated by box plots. In addition, in order to investigate the expression characteristics of prognostic genes in different molecular subtypes of BLCA, the R packages 'ConsensusMIBC' (version 1.1.0) and 'BLCAsubtyping' (version 2.1.1) were used to classify BLCA patients into different molecular subtypes (basal and luminal). The Wilcoxon test was used for the expression levels of prognostic genes between subtypes (p < 0.05). Besides, the immunohistochemically stained landscape of prognostic genes in urinary bladder and urothelial cancer was acquired from the Human Protein Atlas (HPA, https://www.proteinatlas.org/) database.

### 4.14 Gene set enrichment analysis (GSEA) and mutational landscape characterization betwixt two risk teams

Firstly, the GSEA was conducted through the 'clusterProfiler' and 'org.Hs.eg.db' (v 3.13.0) packages in R to compare the variations in the KEGG signaling pathway between the two risk teams (|NES| > 1, NOM p < 0.05 and q < 0.25). The background gene sets, c5.go.v7.4.entrez.gmt (GO) and c2.cp.kegg.v7.4.entrez.gmt (KEGG), were retrieved from the GSEA website (http://www.gsea-msigdb.org/gsea/msigdb). The R package 'maftools' (v 2.12.0)^45^ was employed to characterize the tumor mutation status between two risk teams by calculating Somatic mutation information in the TCGA-BLCA dataset. Besides, the discrepancies of TMB between the two risk teams were computed and compared by the Wilcoxon rank-sum test (p-value < 0.05). We proceeded to investigate the mutation rates of several frequently mutated genes, including TP53, EGFR, FGFR, and KDM6A, in both the high-risk and low-risk teams.

### 4.15 Immune cell infiltration (ICI) and drug sensitivity analyses

The characterization of ICI could indicate the prognosis of BLCA patients. We used the CIBERSORT algorithm to calculate the proportions of 22 immune cells between two risk groups. Wilcoxon rank-sum tests (p-value < 0.05) were employed to select discrepant immune cells and differentially expressed immune checkpoints between the two groups. Immune checkpoints represented a category of immunosuppressive molecules expressed on immune cells, regulating the extent of immune activation. Spearman's correlation analysis was conducted to explore the relationships between discrepant immune cells, differential immune checkpoints, and prognostic genes. Additionally, the Estimation algorithm was utilized to assess the discrepancies in stromal score, immune score, and ESTIMATE score, evaluating immune and stromal components in BLCA tumor tissue. Infiltrating stromal and immune cells are a major component of normal cells in tumour tissues, interfere with tumour signalling in molecular studies and have an important role in tumour biology.

Chemotherapy was commonly used in the clinical treatment of malignant tumours. In this study, 198 kinds of drugs were gathered from the Genomics of Drug Sensitivity in Cancer (GDSC) database (https://www.cancerrxgene.org/) and the half-maximal inhibitory concentration (IC50) was assessed for each patient using the R package oncoPredict (version 0.2). Subsequently, the top 5 sensitive drugs were selected in the high/low-risk teams by ranking them according to logFC and their correlations with risk score were assessed. The drugs searched in GDSC and Cancer Therapeutics Response Portal (CTRP) databases simultaneously were evaluated for their correlations with risk scores.

### 4.16 Statistical analysis

The R program (v 4.2.2) was responsible for bioinformatics analysis in this study. The survival differences were analyzed by the Log-rank test. The inter-group discrepancies were contrasted by the Wilcoxon rank-sum test, and a p-value below 0.05 was regarded to be statistically significant.

## 5. Conclusion

In summary, this study identifies CTSE, XAGE2, MAP1A, CASQ2, and FXYD6 as pivotal prognostic biomarkers for BLCA. The establishment of a risk model and a nomogram model based on these genes provides a novel and effective tool for predicting the clinical outcome of BLCA patients. These findings are expected to contribute significantly to the prognostic evaluation and personalized treatment strategies for individuals with BLCA.

## Supplementary Material

Supplementary figures.

## Figures and Tables

**Table 1:** Cellular filtration for each sample

Sample	Rawdata	ByLibSize	ByFeature	ByMT	ByDoubletScore	Remaining	percent
GSM4006644	3272	52	52	0	165	3003	0.918
GSM4006645	5177	156	156	0	246	4619	0.892
GSM4006646	3157	50	50	0	103	2954	0.936
GSM4006647	5313	160	160	0	226	4767	0.897
GSM4006648	9350	528	526	0	721	7575	0.810
GSM4751267	6519	283	283	0	301	5652	0.867
GSM4751268	5696	172	172	0	288	5064	0.889
GSM5329919	6360	278	278	0	363	5441	0.856

**Table 2:** Marker genes of different cell types

Cell Type	Cluster	Genes
Urothelial cells	Cluster0, Cluster1, Cluster2, Cluster3, Cluster4, Cluster5, Cluster7, Cluster8	EPCAM, KRT8, KRT18
Fibroblasts	Cluster6	PDPN, TAGLN, CD44
Myeloid/macrophage	Cluster9, Cluster11	CD14, CSF1R, AIF1
T cells	Cluster10	CD2, CD3D, CD3E
Endothelial cells	Cluster12	PECAM1, FLT1, A2M

**Table 3:** Expression of key genes in different cell types

Gene	avg_log2FC	cluster	Regulation
ABHD11	-0.267794688	urothelial cells	DOWN
BIN1	0.337796024	myeloid/macrophage	UP
CALD1	0.646281636	fibroblasts	UP
CALD1	-1.101214915	myeloid/macrophage	DOWN
CFD	-0.466170807	endothelial cells	DOWN
CFD	0.354230015	fibroblasts	UP
CFD	-0.466170807	T cells	DOWN
FABP5	0.436666572	myeloid/macrophage	UP
FILIP1L	0.410392469	fibroblasts	UP
GSN	0.602132628	fibroblasts	UP
GSN	-0.301609858	urothelial cells	DOWN
ISYNA1	0.341101688	fibroblasts	UP
LPCAT4	-0.366323428	urothelial cells	DOWN
MYH11	0.863408357	fibroblasts	UP
OLFML3	0.551111708	myeloid/macrophage	UP
S100A14	-0.399176017	myeloid/macrophage	DOWN
TGFB1I1	0.642138789	fibroblasts	UP
TINAGL1	0.66843065	fibroblasts	UP
TINAGL1	-0.29114086	myeloid/macrophage	DOWN
TPM1	0.885118002	fibroblasts	UP
TPM1	-0.693462753	myeloid/macrophage	DOWN

**Table 4:** The information and functions of five prognostic genes encoded proteins

Gene	Ensembl	Protein name	Gene Ontology	Location	Diseases
XAGE2	ENSG00000155622	X Antigen Family Member 2	Enables protein binding	Nucleus, Cytoskeleton, Extracellular	Germ Cell Tumor, Sarcoma
MAP1A	ENSG00000166963	Microtubule Associated Protein 1A	Enables actin binding, Enables protein binding	Cytosol, Nucleus, Cytoskeleton	Macular Degeneration, Age-Related, and Amyotrophic Lateral Sclerosis
CASQ2	ENSG00000118729	Calsequestrin 2	Enables calcium ion binding, Enables protein binding	Endoplasmic reticulum, Cytosol, Mitochondrion	Ventricular Tachycardia, Catecholaminergic Polymorphic, and Catecholaminergic Polymorphic Ventricular Tachycardia
FXYD6	ENSG00000137726	FXYD Domain Containing Ion Transport Regulator 6	Enables molecular_function, Enables protein binding	Plasma membrane	Hypomagnesemia, Renal
CTSE	ENSG00000196188	Cathepsin E	Enables aspartic-type endopeptidase activity, Enables peptidase activity	Endosome, Lysosome	Langerhans Cell Histiocytosis and Gastric Papillary Adenocarcinoma
