# Supplementary material for: Integrative multi-omics and machine learning identify a robust signature for discriminating prognosis and therapeutic targets in bladder cancer
Source: J Cancer. 2025 Jan 27;16(5):1479–503. doi: 10.7150/jca.105066 (PMC11843231; doi:10.7150/jca.105066)
Supplement: Supplementary file 1 — Supplementary figures. [file jcav16p1479s1.pdf]

Supplementary Figures

Fig.S1: Processing of transcriptomic, proteomic and acetylomic sequencing data.

Principal component analysis for transcriptomics **(a)**, proteomics **(b)**, and acetylomics **(c)** sequencing data.

Fig.S2: Volcano plots for DEGs and DEPs.

(a) Volcano plot of DEGs between BLCA and controls in transcriptomic sequencing dataset. **(b)** Volcano plot of DEPs between BLCA and controls in proteomics dataset.

Fig.S3: GO and KEGG enrichment analysis of DEGs, DEPs and DEPAs.

GO enrichment analysis showing the biological processes (BP), cellular component (CC), and molecular function (MF) enriched by DEGs **(a)**, DEPs **(c)**, DEPAs **(e)**. KEGG enrichment analysis displaying pathways involved in DEGs **(b)**, DEPs **(d)**, DEPAs **(f)**.

Fig.S4: Intersection of DEGs and DEPs with the same expression trends.

(a) Intersection of up-regulated DEGs and DEPs to obtain up-regulated intersecting genes. **(b)** Venn diagram of down-regulated DEGs and DEPs to acquire down-regulated intersecting genes.

Fig.S5: RNA-Seq data quality control and sample characterisation.

(a, b) The number of nFeature RNA and nCount RNA after quality control. **(c)** Principal component analysis for the samples. **(d)** Elbowplot for identifying the optimal principal components. The proportion of 5 cell types in samples **(e)**, as well as BLCA tumor tissue and control groups **(f)**.

Fig.S6: Screening of intercellular hypervariable genes.

Fig.S7: Circle plot of correlations between key genes.

Fig.S8: Heatmap of cell cluster ratios of fibroblasts and myeloid macrophages in BLCA tumour tissue versus controls.

Heat maps revealing the proportion of 7 cell clusters formed by fibroblasts **(a)** and myeloid macrophages **(b)**. The proportion of 7 cell clusters formed by fibroblasts **(c)** and myeloid macrophages **(d)** in BLCA tumor tissue and control groups respectively.

Fig.S9: Expression of key genes in different branches of fibroblasts and myeloid macrophages and number of ligand-receptor pairs for cell-cell interactions.

Expression of pivotal genes in different branches of fibroblasts **(a)** and myeloid macrophages **(b)**. **(c)** The number of ligand-receptor pairs for cell-cell interactions.

Fig.S10: Analysis of differentially expressed genes associated with BLCA between the two clusters.

Volcano plot **(a)** and heat map **(b)** of Cluster-DEGs between 2 clusters. Identification of GO **(c)** and KEGG **(d)** entries involved by Cluster-DEGs. Volcano plot **(e)** and heat map **(f)** of BLCA-DEGs between BLCA and controls in TCGA-BLCA dataset.

Fig.S11: PH assumptions for verifying the applicability of the univariate Cox regression model.

Fig.S12: Validation of risk models in GSE13507 dataset.

Risk curve **(a)** and K-M curve **(b)** predicting the overall survival and survival probability of high- and low-risk patients in GSE13507 dataset. **(c)** ROC curves to evaluate the predictive performance of risk model in GSE13507 dataset.

Fig.S13: The sankey plot showing the flow between different clinicopathological features.

Fig.S14: K-M survival stratification analyses.

The differences in survival probability of age $\leq$ 60 years **(a)**, age > 60 years **(b)**; pathological T1 + T2 **(c)**, pathological T3 + T4 **(d)**; pathological N0 **(e)**; pathological N1 **(f)**.

Fig.S15: Comparison of TMB differences and mutation rates of TP53 and KDM6A genes between high and low risk groups.

(a) The TMB differences between high- and low-risk teams. The mutation rate of TP53 **(b)** and KDM6A **(c)** between high- and low-risk teams.

Fig.S16: Lollipop plots depicting immune cell relationships with CASQ2, CTSE, FXYD6, MAP1A, and XAGE2.

Lollipop polts illustrating the relationships between discrepant immune cells and CASQ2 **(a)**, CTSE **(b)**, FXYD6 **(c)**, MAP1A **(d)** and XAGE2 **(e)**.

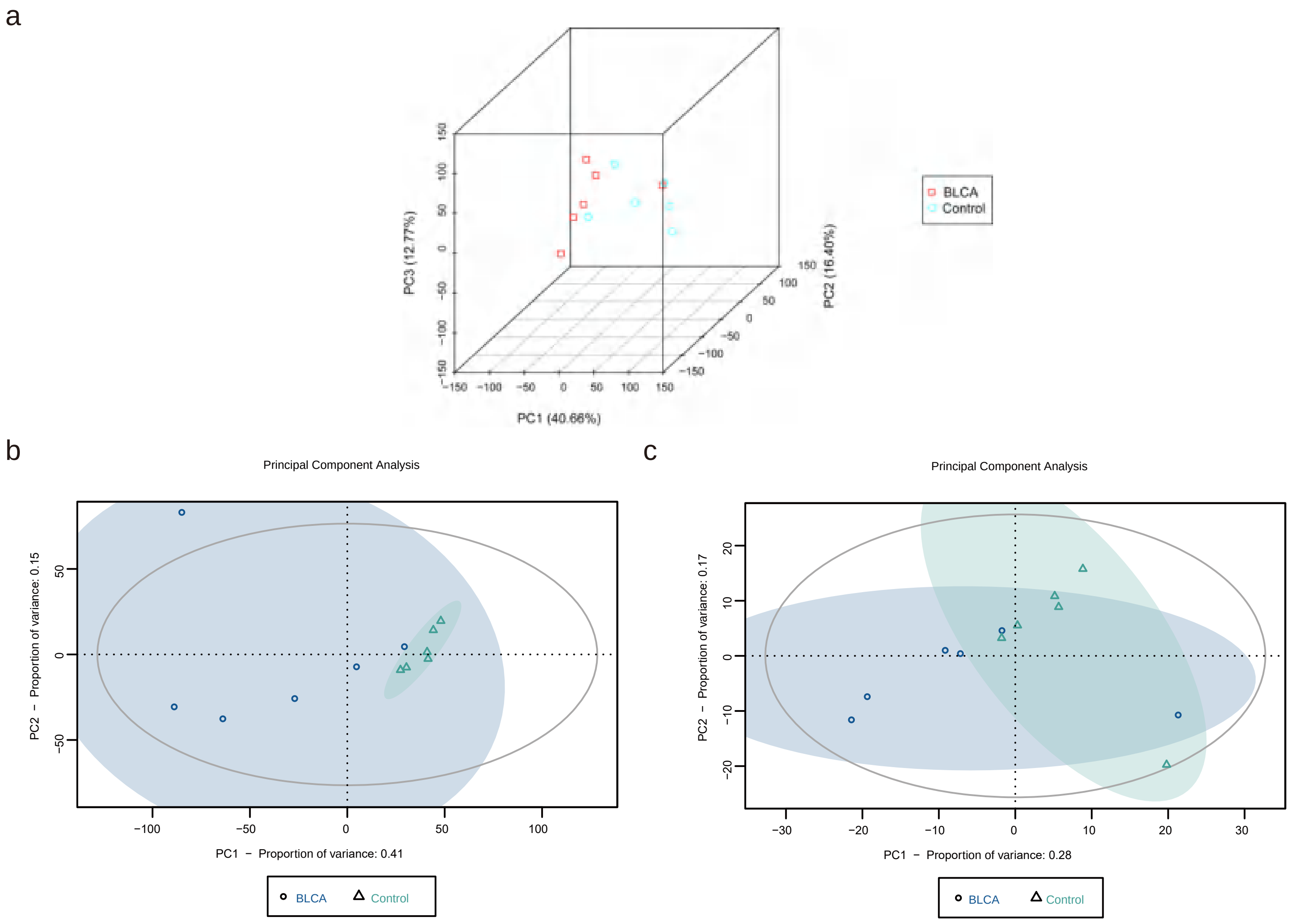

a
PC1 (40.66%)
PC2 (16.40%)
PC3 (12.77%)
BLCA
Control
b
Principal Component Analysis
PC1 − Proportion of variance: 0.41
PC2 − Proportion of variance: 0.15
BLCA
Control
c
Principal Component Analysis
PC1 − Proportion of variance: 0.28
PC2 − Proportion of variance: 0.17
BLCA
Control

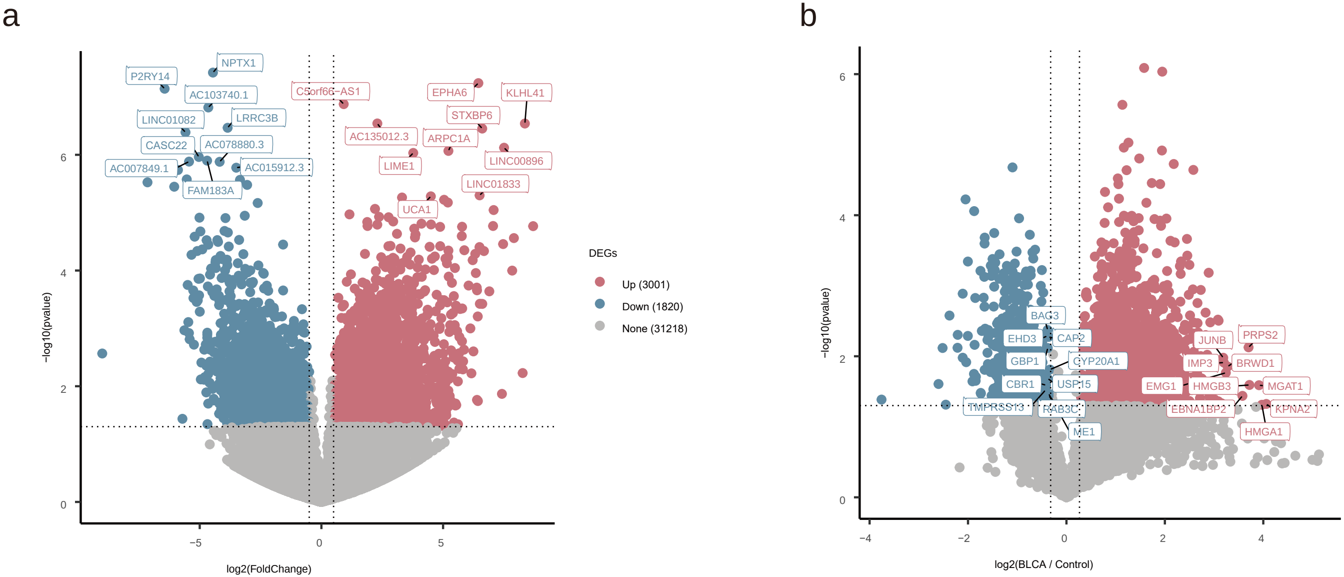
a
b
NPTX1
P2RY14
AC103740.1
LINC01082
LRRC3B
CASC22
AC078880.3
AC007849.1
AC015912.3
FAM183A
C5orf66−AS1
EPHA6
KLHL41
STXBP6
AC135012.3
ARPC1A
LIME1
LINC00896
LINC01833
UCA1
−log10(pvalue)
log2(FoldChange)
DEGs
Up (3001)
Down (1820)
None (31218)
BAG3
EHD3
CAP2
GBP1
CYP20A1
CBR1
USP15
TMPRSS13
RAB3C
ME1
PRPS2
JUNB
IMP3
BRWD1
EMG1
HMGB3
MGAT1
EBNA1BP2
KPNA2
HMGA1
log2(BLCA / Control)
DEPs
Up (535)
Down (1860)
None (3890)

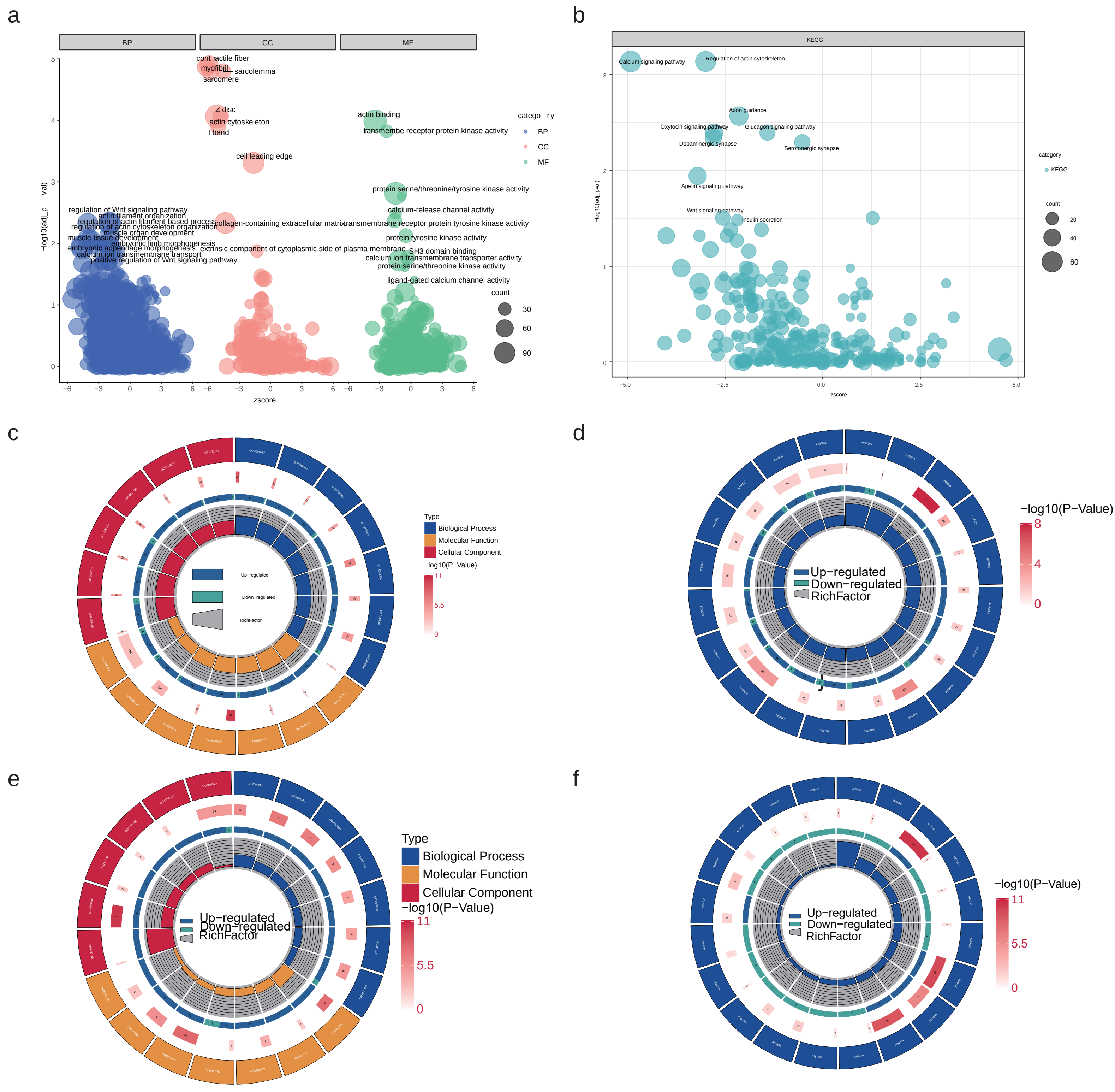

a
BP
CC
MF
contractile fiber
myofibril
sarcolemma
sarcomere
Z disc
actin cytoskeleton
I band
cell leading edge
actin binding
transmembrane receptor protein kinase activity
protein serine/threonine/tyrosine kinase activity
calcium-release channel activity
transmembrane receptor protein tyrosine kinase activity
protein tyrosine kinase activity
SH3 domain binding
calcium ion transmembrane transporter activity
protein serine/threonine kinase activity
ligand-gated calcium channel activity
regulation of Wnt signaling pathway
actin filament organization
regulation of actin filament-based process
regulation of actin cytoskeleton organization
muscle organ development
muscle tissue development
embryonic limb morphogenesis
embryonic appendage morphogenesis
calcium ion transmembrane transport
positive regulation of Wnt signaling pathway
collagen-containing extracellular matrix
extrinsic component of cytoplasmic side of plasma membrane
category
BP
CC
MF
count
30
60
90
−log10(adj_pval)
zscore
b
KEGG
Calcium signaling pathway
Regulation of actin cytoskeleton
Axon guidance
Oxytocin signaling pathway
Glucagon signaling pathway
Dopaminergic synapse
Serotonergic synapse
Apelin signaling pathway
Wnt signaling pathway
Insulin secretion
category
KEGG
count
20
40
60
−log10(adj_pval)
zscore
c
Up-regulated
Down-regulated
RichFactor
Type
Biological Process
Molecular Function
Cellular Component
−log10(P−Value)
11
5.5
0
d
Up−regulated
Down−regulated
RichFactor
−log10(P−Value)
8
4
0
e
Up−regulated
Down−regulated
RichFactor
Type
Biological Process
Molecular Function
Cellular Component
−log10(P−Value)
11
5.5
0
f
Up−regulated
Down−regulated
RichFactor
−log10(P−Value)
11
5.5
0

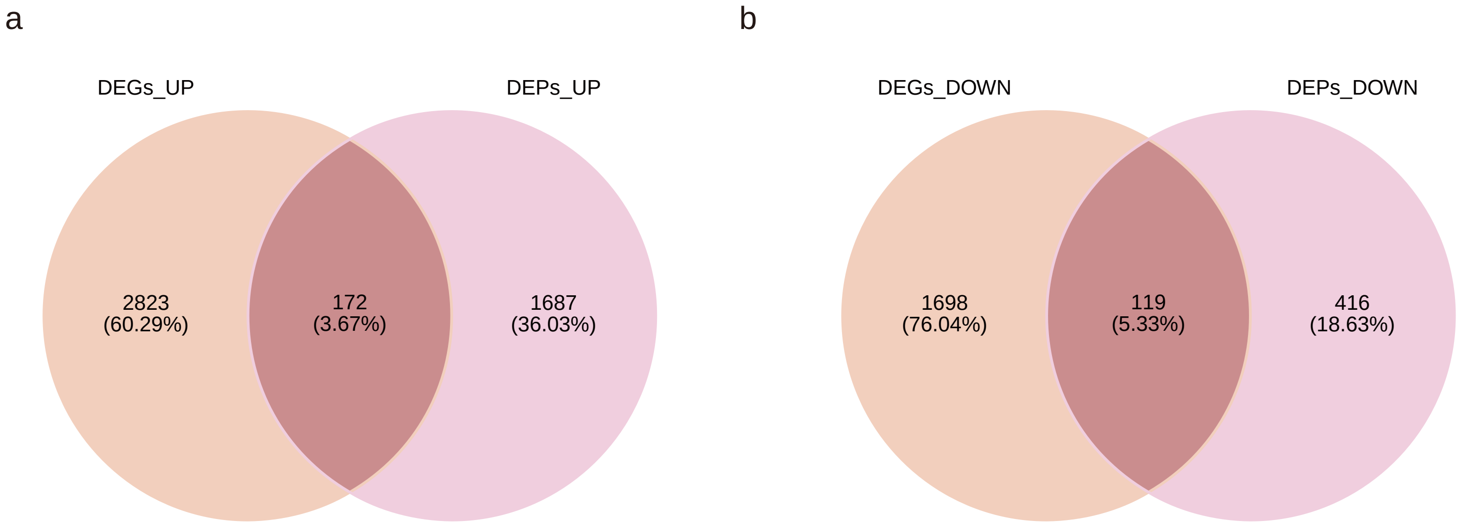
a
DEGs_UP
DEPs_UP
2823
(60.29%)
172
(3.67%)
1687
(36.03%)
b
DEGs_DOWN
DEPs_DOWN
1698
(76.04%)
119
(5.33%)
416
(18.63%)

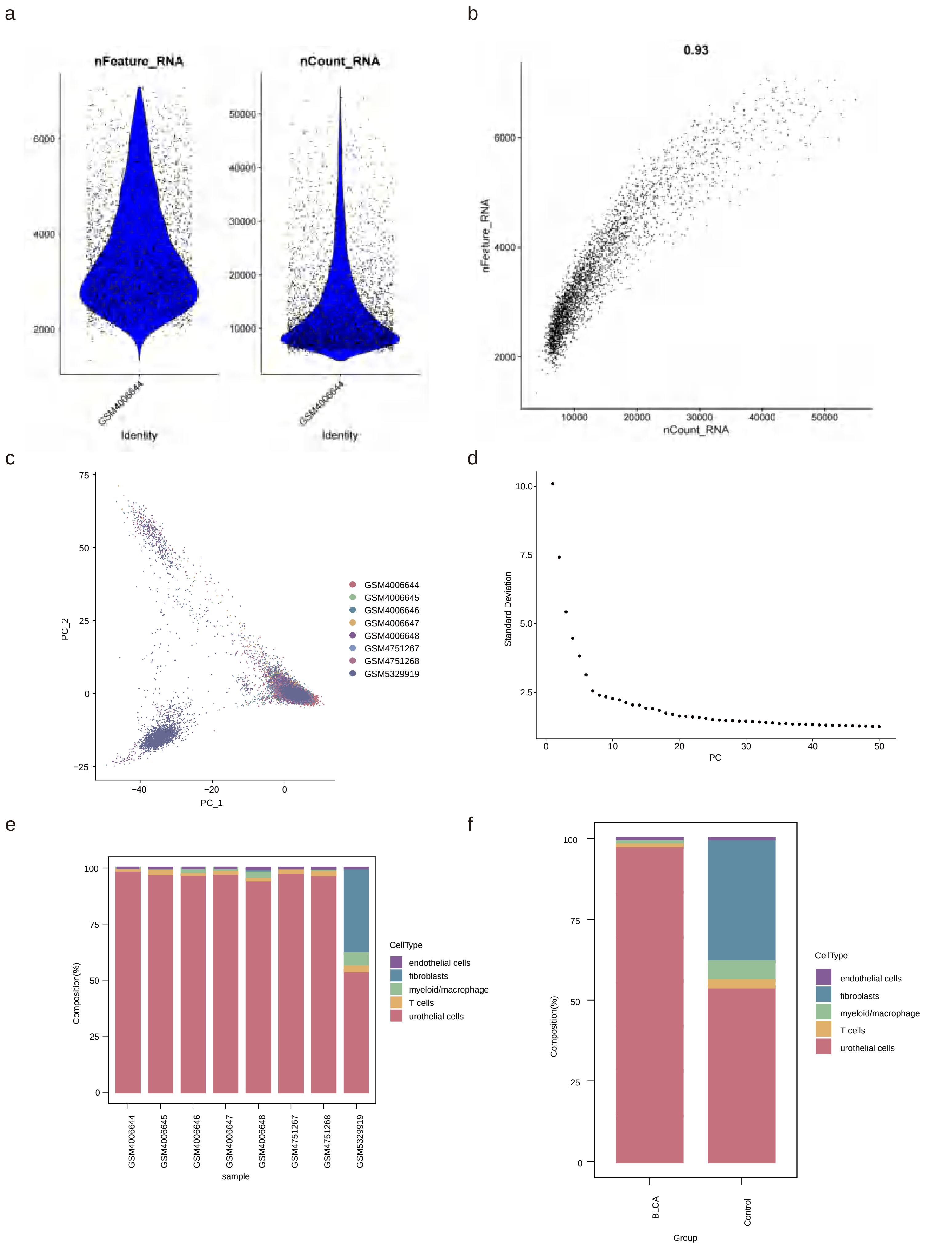
a
nFeature_RNA
nCount_RNA
6000
4000
2000
50000
40000
30000
20000
10000
GSM4006644
GSM4006644
Identity
Identity
b
0.93
nFeature_RNA
6000
4000
2000
10000
20000
30000
40000
50000
nCount_RNA
c
75
50
25
0
−25
PC_2
−40
−20
0
PC_1
GSM4006644
GSM4006645
GSM4006646
GSM4006647
GSM4006648
GSM4751267
GSM4751268
GSM5329919
d
10.0
7.5
5.0
2.5
Standard Deviation
0
10
20
30
40
50
PC
e
100
75
50
25
0
Composition(%)
GSM4006644
GSM4006645
GSM4006646
GSM4006647
GSM4006648
GSM4751267
GSM4751268
GSM5329919
sample
CellType
endothelial cells
fibroblasts
myeloid/macrophage
T cells
urothelial cells
f
100
75
50
25
0
Composition(%)
BLCA
Control
Group
CellType
endothelial cells
fibroblasts
myeloid/macrophage
T cells
urothelial cells

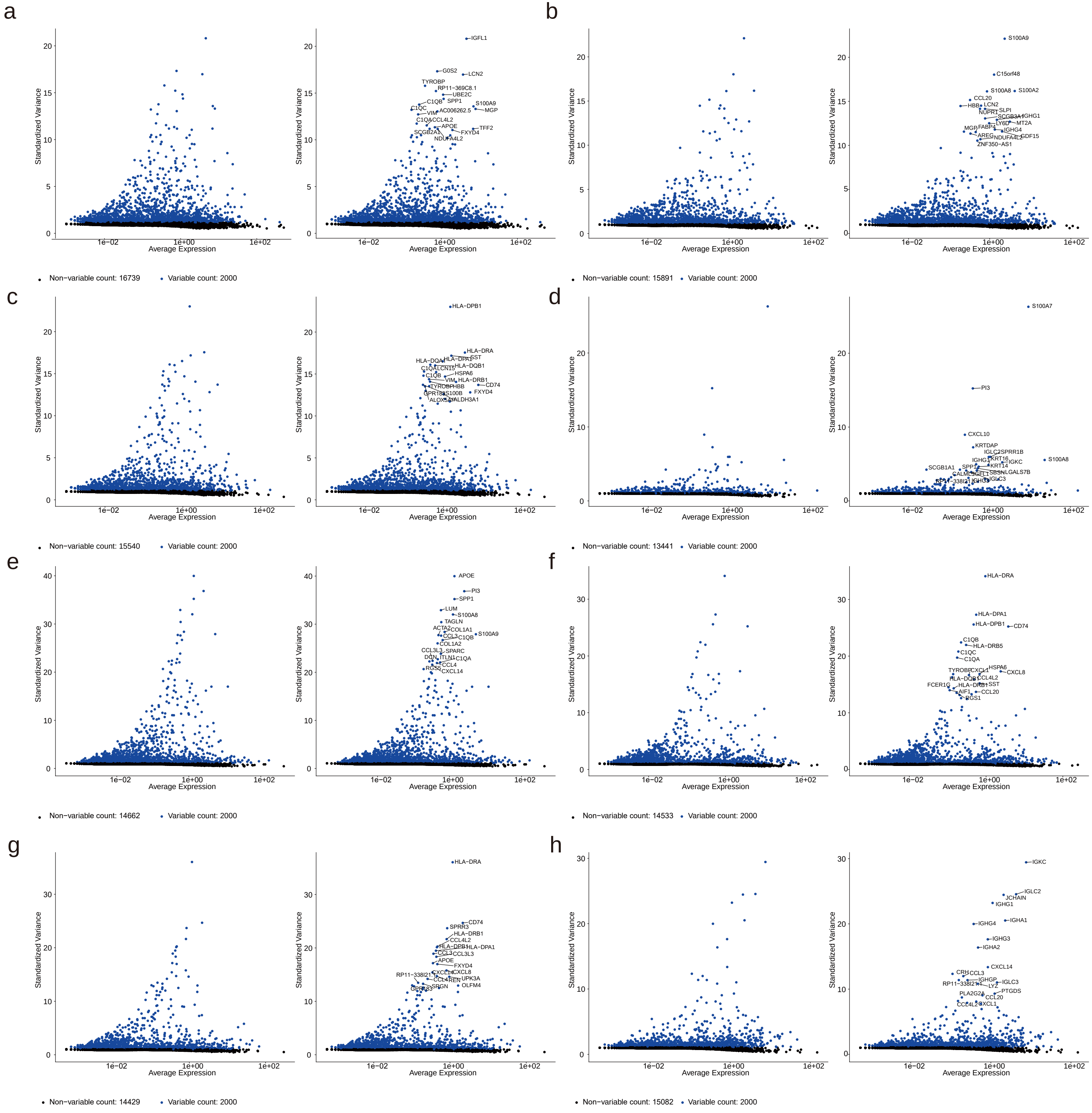
a
b
c
d
e
f
g
h
Standardized Variance
Average Expression
1e-02
1e+00
1e+02
Non−variable count: 16739
Variable count: 2000
Non−variable count: 15891
Non−variable count: 15540
Non−variable count: 13441
Non−variable count: 14662
Non−variable count: 14533
Non−variable count: 14429
Non−variable count: 15082
IGFL1
G0S2
LCN2
TYROBP
RP11−369C8.1
UBE2C
C1QB
SPP1
S100A9
C1QC
MGP
VIM
AC006262.5
C1QA
CCL4L2
APOE
TFF2
SCGB2A1
FXYD4
NDUFA4L2
S100A9
C15orf48
S100A8
S100A2
CCL20
HBB
LCN2
SLPI
NUPR1
SCGB3A1
IGHG1
LY6D
MT2A
FABP4
MGP
IGHG4
AREG
NDUFA4L2
GDF15
ZNF350−AS1
HLA−DPB1
HLA−DRA
SST
HLA−DQA1
HLA−DPA1
HLA−DQB1
HSPA6
VIM
HLA−DRB1
CD74
FXYD4
ALDH3A1
S100A7
PI3
CXCL10
KRTDAP
SPRR1B
S100A8
KRT16
IGKC
KRT14
SCGB1A1
LGALS7B
APOE
PI3
SPP1
LUM
S100A8
TAGLN
ACTA2
COL1A1
C1QB
S100A9
COL1A2
SPARC
C1QA
CCL4
CXCL14
HLA−DRA
HLA−DPA1
HLA−DPB1
CD74
C1QB
HLA−DRB5
C1QC
C1QA
HSPA6
CXCL8
CCL4L2
SST
FCER1G
CCL20
RGS1
HLA−DRA
CD74
SPRR3
HLA−DRB1
CCL4L2
HLA−DPA1
CCL3L3
APOE
FXYD4
CXCL8
UPK3A
OLFM4
SRGN
IGKC
IGLC2
JCHAIN
IGHG1
IGHA1
IGHG4
IGHG3
IGHA2
CXCL14
CCL3
IGLC3
LYZ
PTGDS
CCL20
CXCL1

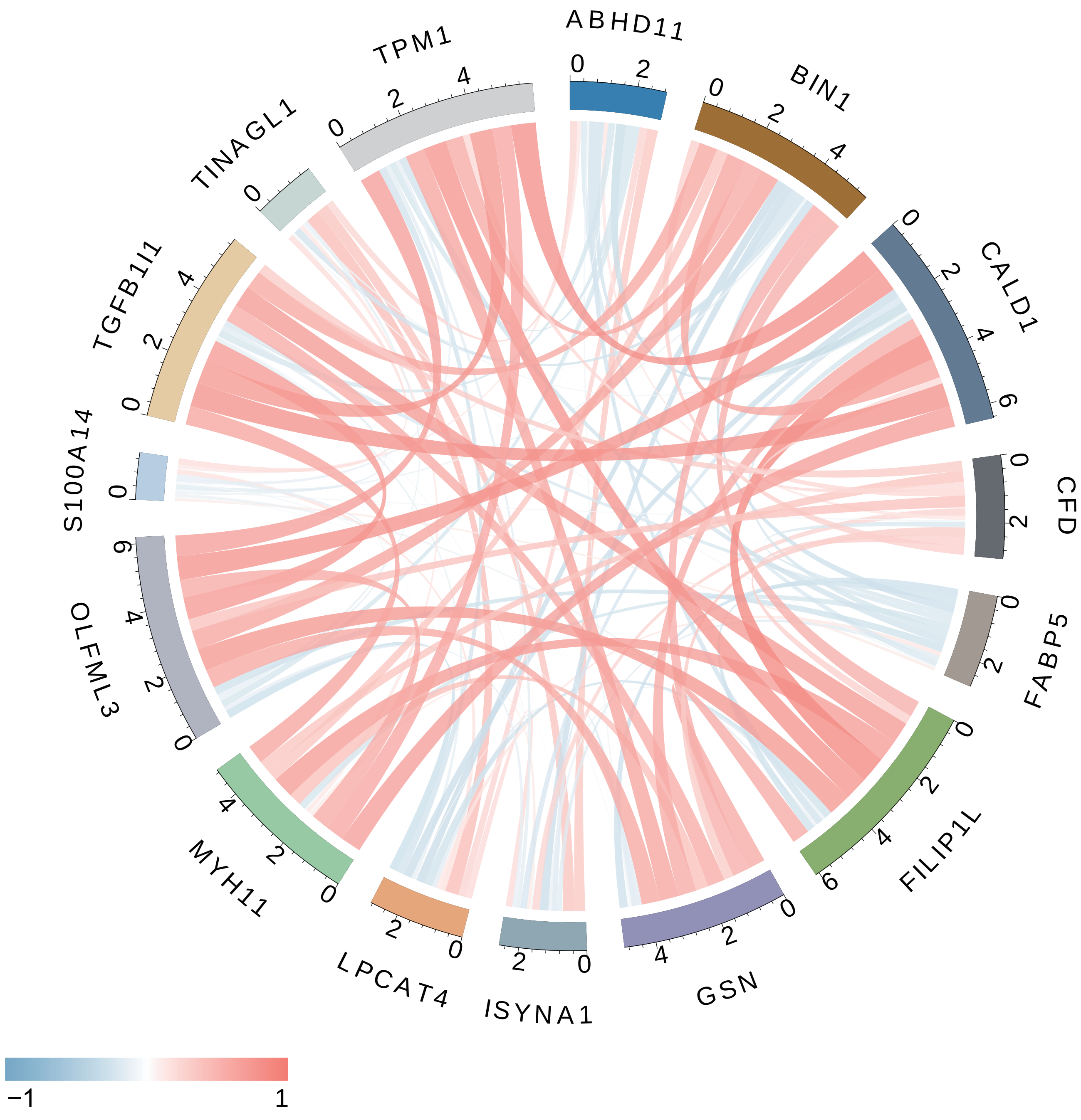

ABHD11
BIN1
CALD1
CFD
FABP5
FILIP1L
GSN
ISYNA1
LPCAT4
MYH11
OLFML3
S100A14
TGFB1I1
TINAGL1
TPM1
−1
1

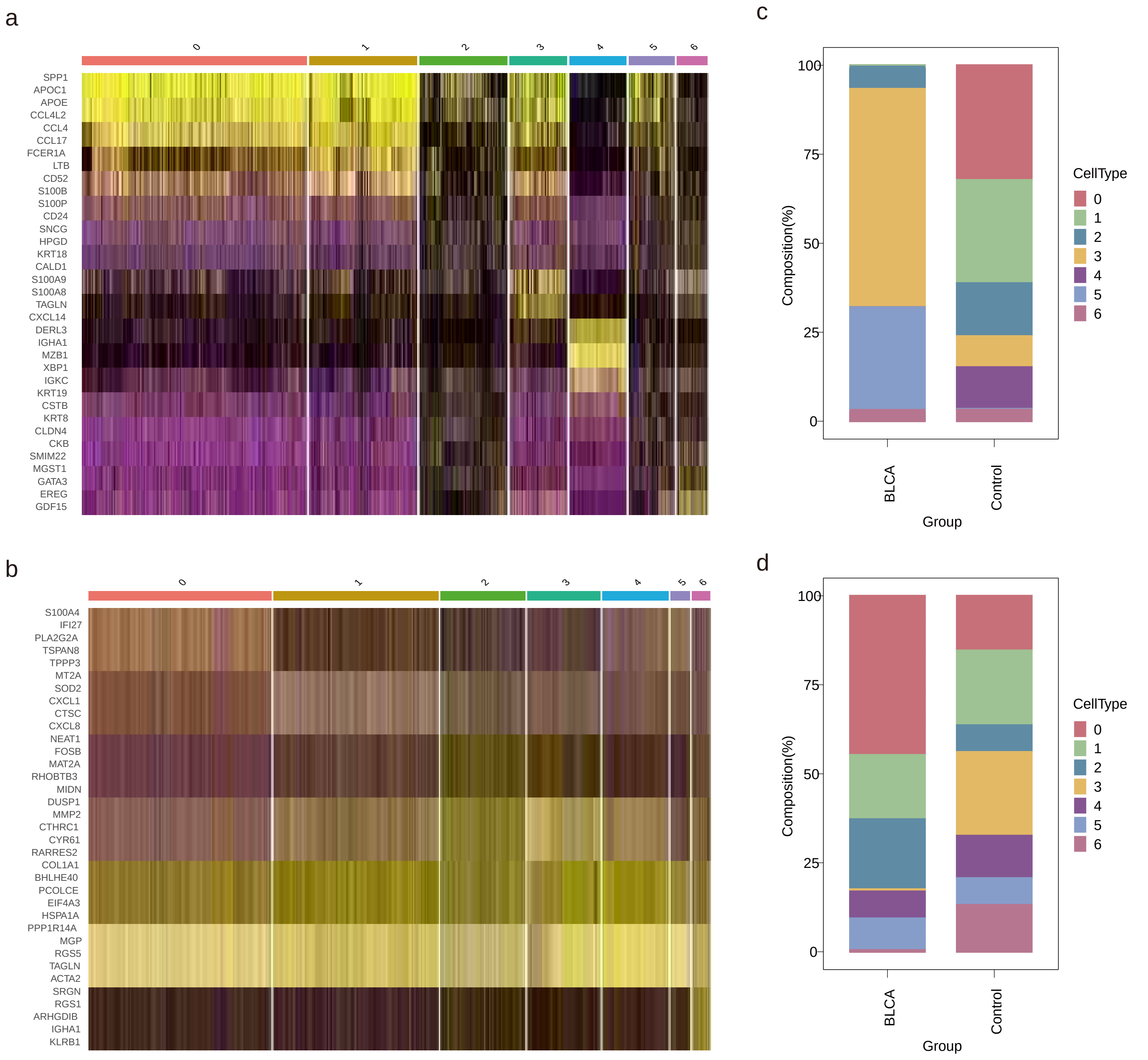

a
0
1
2
3
4
5
6
SPP1
APOC1
APOE
CCL4L2
CCL4
CCL17
FCER1A
LTB
CD52
S100B
S100P
CD24
SNCG
HPGD
KRT18
CALD1
S100A9
S100A8
TAGLN
CXCL14
DERL3
IGHA1
MZB1
XBP1
IGKC
KRT19
CSTB
KRT8
CLDN4
CKB
SMIM22
MGST1
GATA3
EREG
GDF15
b
S100A4
IFI27
PLA2G2A
TSPAN8
TPPP3
MT2A
SOD2
CXCL1
CTSC
CXCL8
NEAT1
FOSB
MAT2A
RHOBTB3
MIDN
DUSP1
MMP2
CTHRC1
CYR61
RARRES2
COL1A1
BHLHE40
PCOLCE
EIF4A3
HSPA1A
PPP1R14A
MGP
RGS5
TAGLN
ACTA2
SRGN
RGS1
ARHGDIB
IGHA1
KLRB1
c
Composition(%)
100
75
50
25
0
BLCA
Control
Group
CellType
d
Composition(%)
Group
CellType

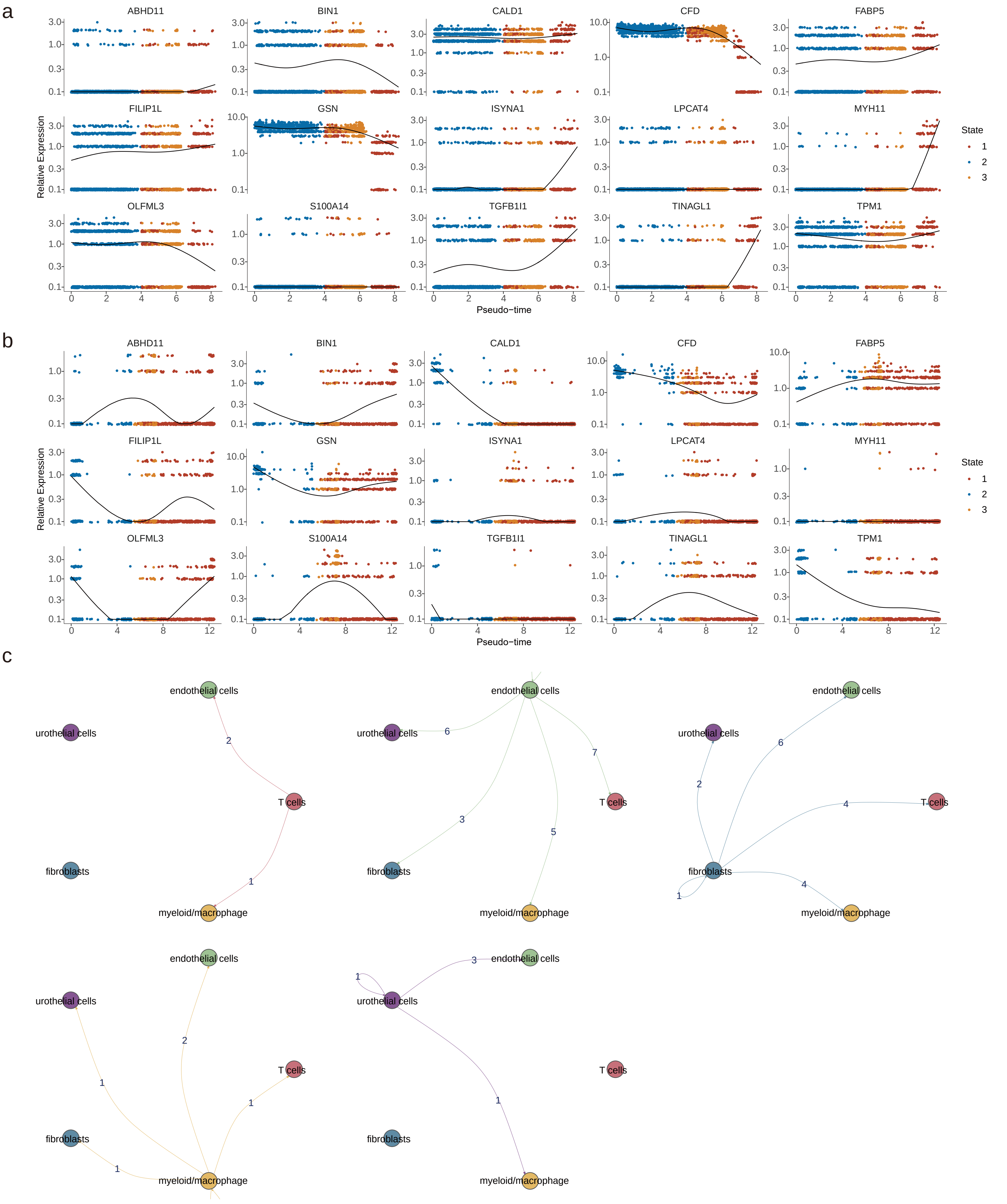
a
ABHD11
BIN1
CALD1
CFD
FABP5
FILIP1L
GSN
ISYNA1
LPCAT4
MYH11
OLFML3
S100A14
TGFB1I1
TINAGL1
TPM1
Relative Expression
Pseudo−time
State
1
2
3
b
ABHD11
BIN1
CALD1
CFD
FABP5
FILIP1L
GSN
ISYNA1
LPCAT4
MYH11
OLFML3
S100A14
TGFB1I1
TINAGL1
TPM1
Relative Expression
Pseudo−time
State
1
2
3
c
endothelial cells
urothelial cells
T cells
fibroblasts
myeloid/macrophage

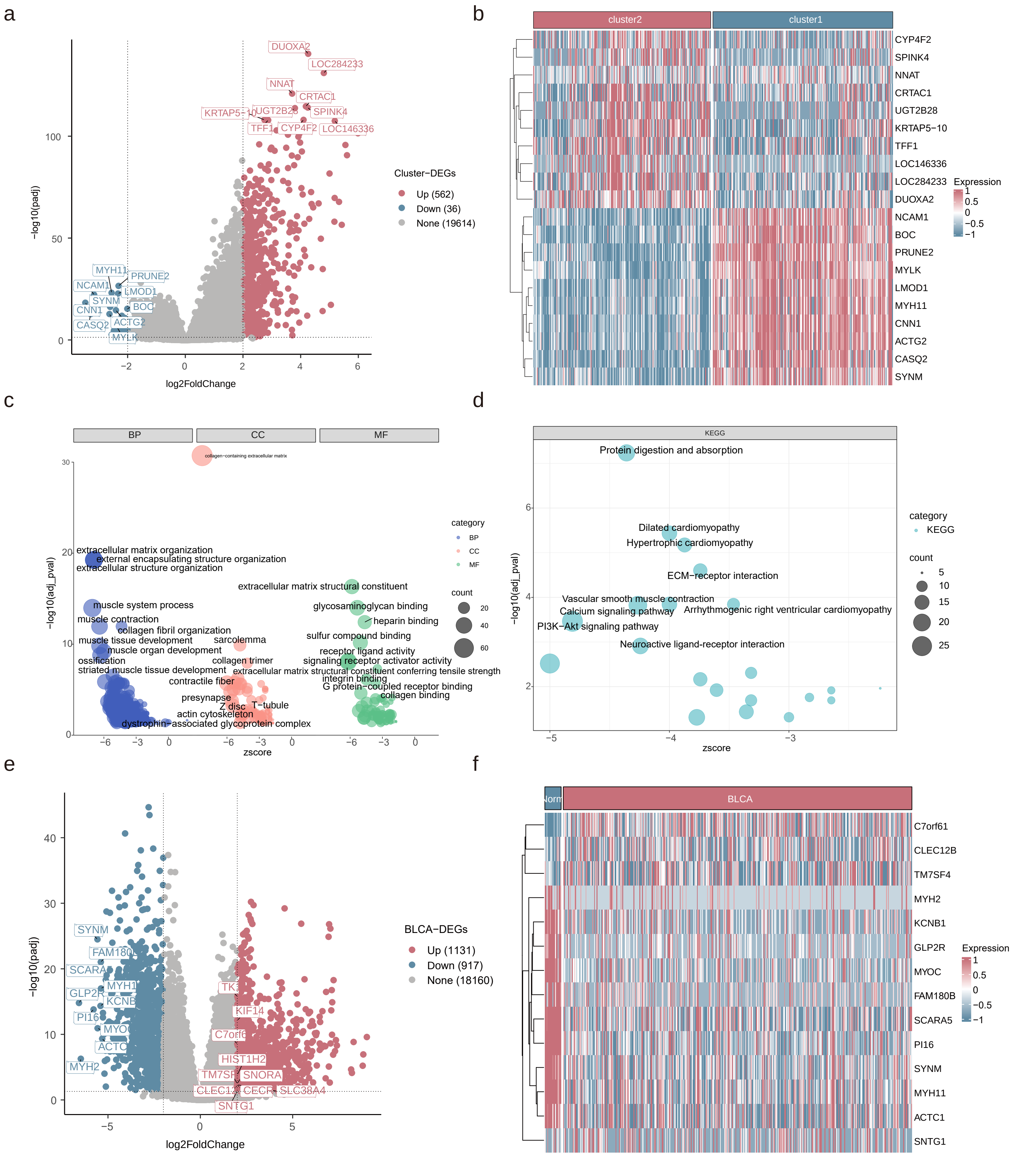
a
−log10(padj)
log2FoldChange
Cluster−DEGs
Up (562)
Down (36)
None (19614)
DUOXA2
LOC284233
NNAT
CRTAC1
KRTAP5−10
UGT2B28
SPINK4
TFF1
CYP4F2
LOC146336
MYH11
PRUNE2
NCAM1
LMOD1
SYNM
CNN1
BOC
CASQ2
ACTG2
MYLK
b
cluster2
cluster1
Expression
CYP4F2
SPINK4
NNAT
CRTAC1
UGT2B28
KRTAP5−10
TFF1
LOC146336
LOC284233
DUOXA2
NCAM1
BOC
PRUNE2
MYLK
LMOD1
MYH11
CNN1
ACTG2
CASQ2
SYNM
c
BP
CC
MF
−log10(adj_pval)
zscore
category
count
collagen−containing extracellular matrix
extracellular matrix organization
external encapsulating structure organization
extracellular structure organization
extracellular matrix structural constituent
muscle system process
glycosaminoglycan binding
muscle contraction
heparin binding
collagen fibril organization
sulfur compound binding
muscle tissue development
sarcolemma
muscle organ development
receptor ligand activity
ossification
collagen trimer
signaling receptor activator activity
striated muscle tissue development
extracellular matrix structural constituent conferring tensile strength
contractile fiber
integrin binding
G protein−coupled receptor binding
collagen binding
presynapse
Z disc
T−tubule
actin cytoskeleton
dystrophin−associated glycoprotein complex
d
KEGG
−log10(adj_pval)
zscore
category
count
Protein digestion and absorption
Dilated cardiomyopathy
Hypertrophic cardiomyopathy
ECM−receptor interaction
Vascular smooth muscle contraction
Calcium signaling pathway
Arrhythmogenic right ventricular cardiomyopathy
PI3K−Akt signaling pathway
Neuroactive ligand-receptor interaction
e
−log10(padj)
log2FoldChange
BLCA−DEGs
Up (1131)
Down (917)
None (18160)
SYNM
FAM180
SCARA
MYH1
GLP2R
KCNB
PI16
MYO
ACTC
MYH2
TK
KIF14
C7orf6
HIST1H2
TM7SF
SNORA
CLEC12
CECR
SLC38A4
SNTG1
f
Norm
BLCA
Expression
C7orf61
CLEC12B
TM7SF4
MYH2
KCNB1
GLP2R
MYOC
FAM180B
SCARA5
PI16
SYNM
MYH11
ACTC1
SNTG1

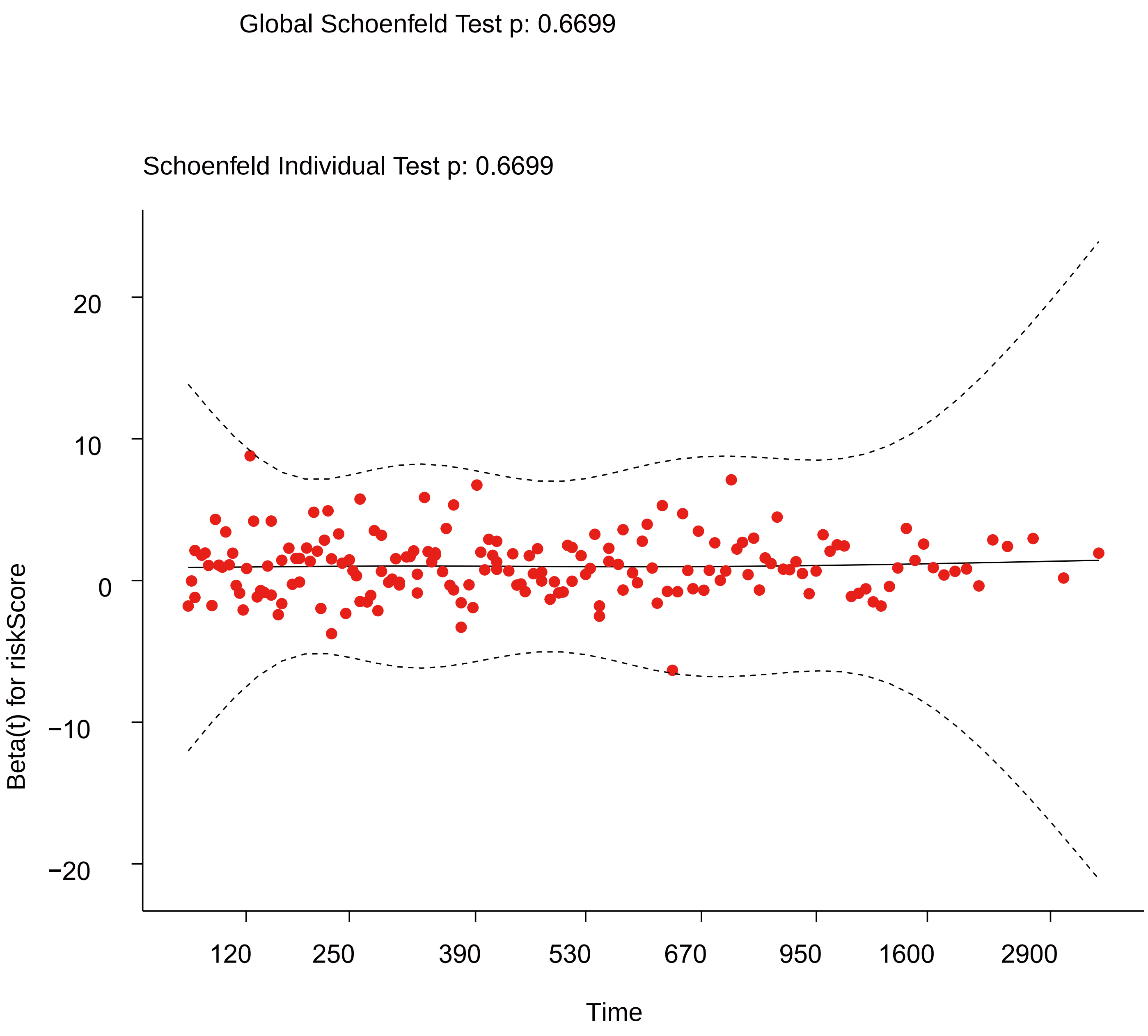
Global Schoenfeld Test p: 0.6699
Schoenfeld Individual Test p: 0.6699
Beta(t) for riskScore
20
10
0
−10
−20
120
250
390
530
670
950
1600
2900
Time

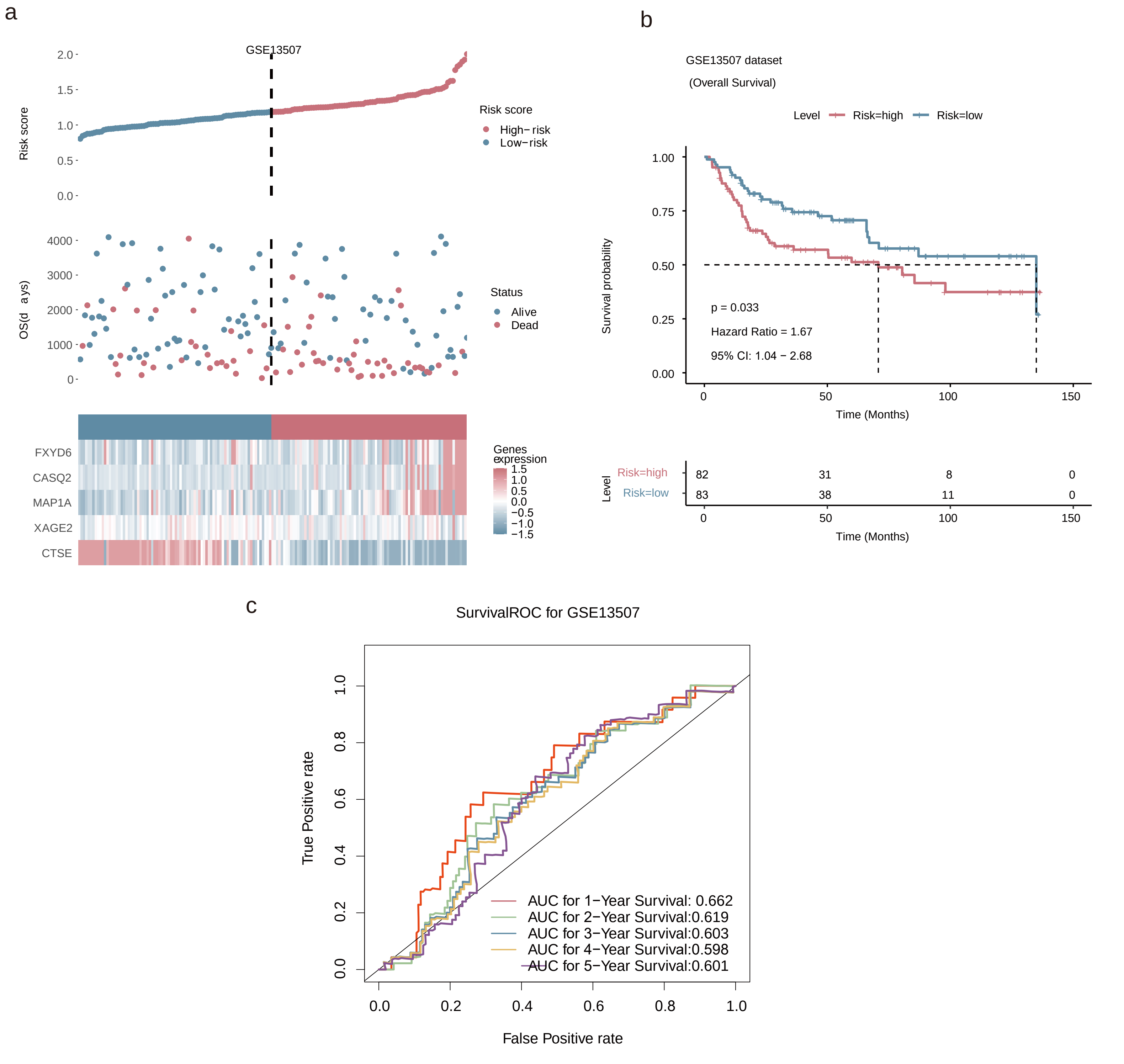
a
GSE13507
Risk score
2.0
1.5
1.0
0.5
0.0
Risk score
High−risk
Low−risk
OS(days)
4000
3000
2000
1000
0
Status
Alive
Dead
FXYD6
CASQ2
MAP1A
XAGE2
CTSE
Genes expression
1.5
1.0
0.5
0.0
−0.5
−1.0
−1.5
b
GSE13507 dataset
(Overall Survival)
Level
Risk=high
Risk=low
Survival probability
1.00
0.75
0.50
0.25
0.00
p = 0.033
Hazard Ratio = 1.67
95% CI: 1.04 − 2.68
0
50
100
150
Time (Months)
Level
Risk=high
Risk=low
82
31
8
0
83
38
11
0
Time (Months)
c
SurvivalROC for GSE13507
True Positive rate
0.0
0.2
0.4
0.6
0.8
1.0
False Positive rate
AUC for 1−Year Survival: 0.662
AUC for 2−Year Survival:0.619
AUC for 3−Year Survival:0.603
AUC for 4−Year Survival:0.598
AUC for 5−Year Survival:0.601

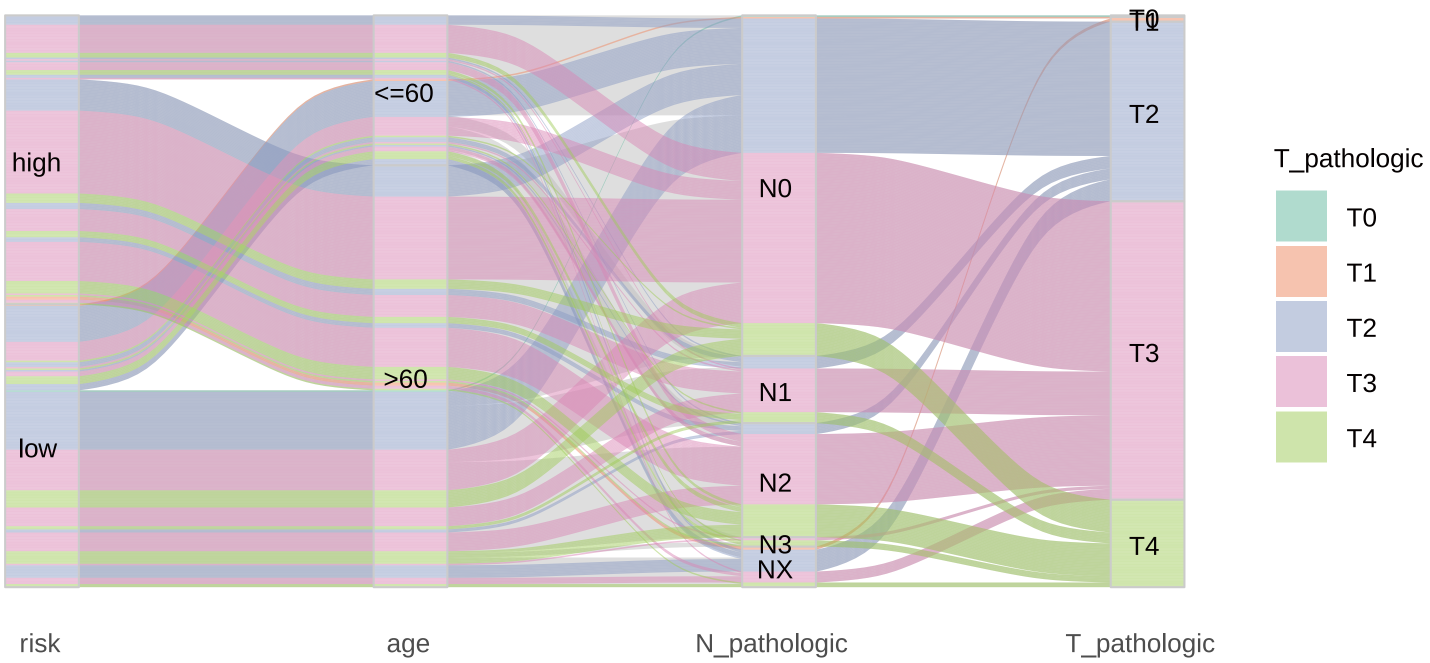

high
low
<=60
>60
N0
N1
N2
N3
NX
T0
T1
T2
T3
T4
risk
age
N_pathologic
T_pathologic
T_pathologic
T0
T1
T2
T3
T4

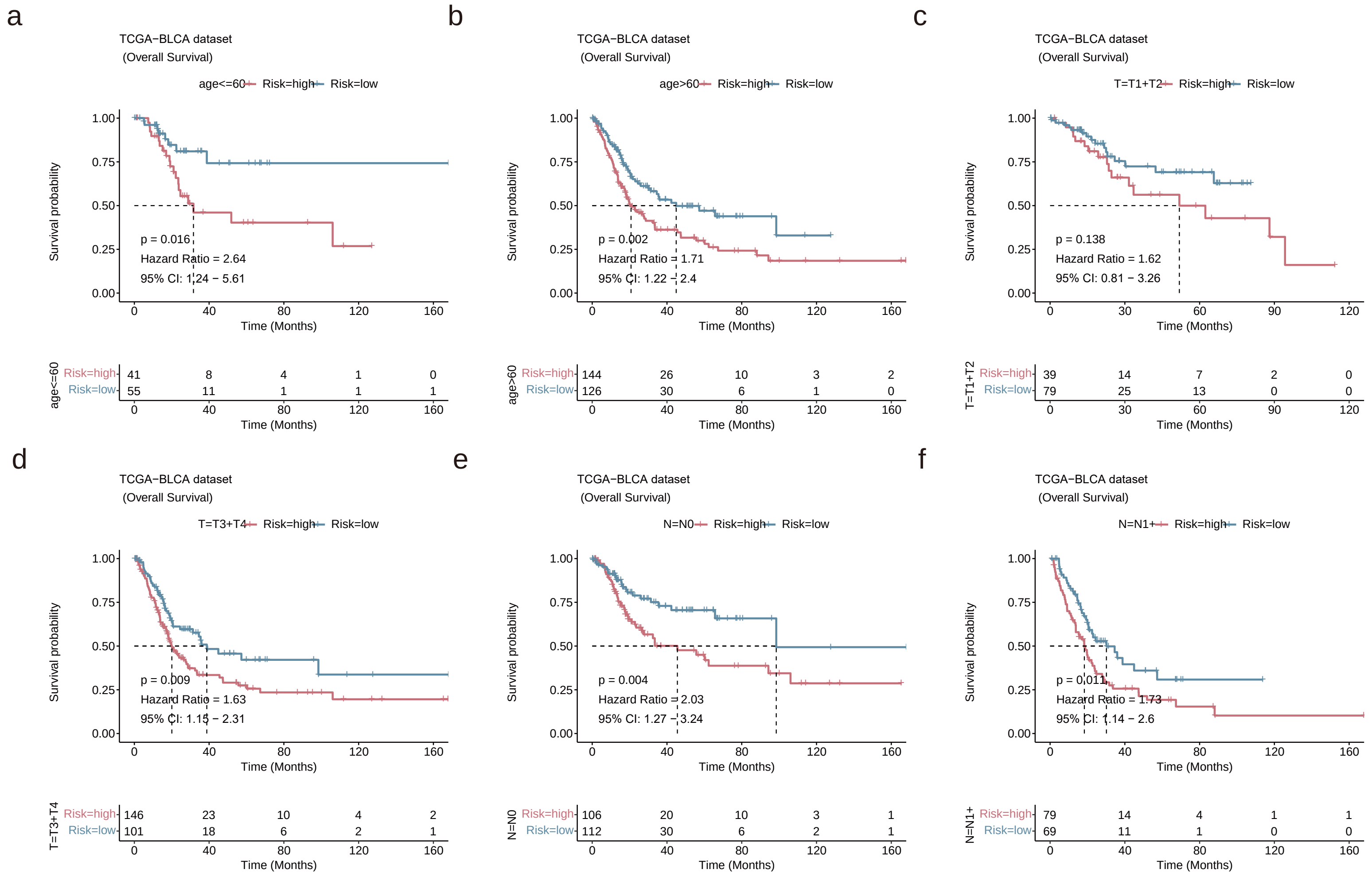
a
TCGA−BLCA dataset
(Overall Survival)
age<=60 Risk=high Risk=low
Survival probability
p = 0.016
Hazard Ratio = 2.64
95% CI: 1.24 − 5.61
Time (Months)
Risk=high 41 8 4 1 0
Risk=low 55 11 1 1 1
b
TCGA−BLCA dataset
(Overall Survival)
age>60 Risk=high Risk=low
p = 0.002
Hazard Ratio = 1.71
95% CI: 1.22 − 2.4
Risk=high 144 26 10 3 2
Risk=low 126 30 6 1 0
c
TCGA−BLCA dataset
(Overall Survival)
T=T1+T2 Risk=high Risk=low
p = 0.138
Hazard Ratio = 1.62
95% CI: 0.81 − 3.26
Risk=high 39 14 7 2 0
Risk=low 79 25 13 0 0
d
TCGA−BLCA dataset
(Overall Survival)
T=T3+T4 Risk=high Risk=low
p = 0.009
Hazard Ratio = 1.63
95% CI: 1.15 − 2.31
Risk=high 146 23 10 4 2
Risk=low 101 18 6 2 1
e
TCGA−BLCA dataset
(Overall Survival)
N=N0 Risk=high Risk=low
p = 0.004
Hazard Ratio = 2.03
95% CI: 1.27 − 3.24
Risk=high 106 20 10 3 1
Risk=low 112 30 6 2 1
f
TCGA−BLCA dataset
(Overall Survival)
N=N1+ Risk=high Risk=low
p = 0.011
Hazard Ratio = 1.73
95% CI: 1.14 − 2.6
Risk=high 79 14 4 1 1
Risk=low 69 11 1 0 0

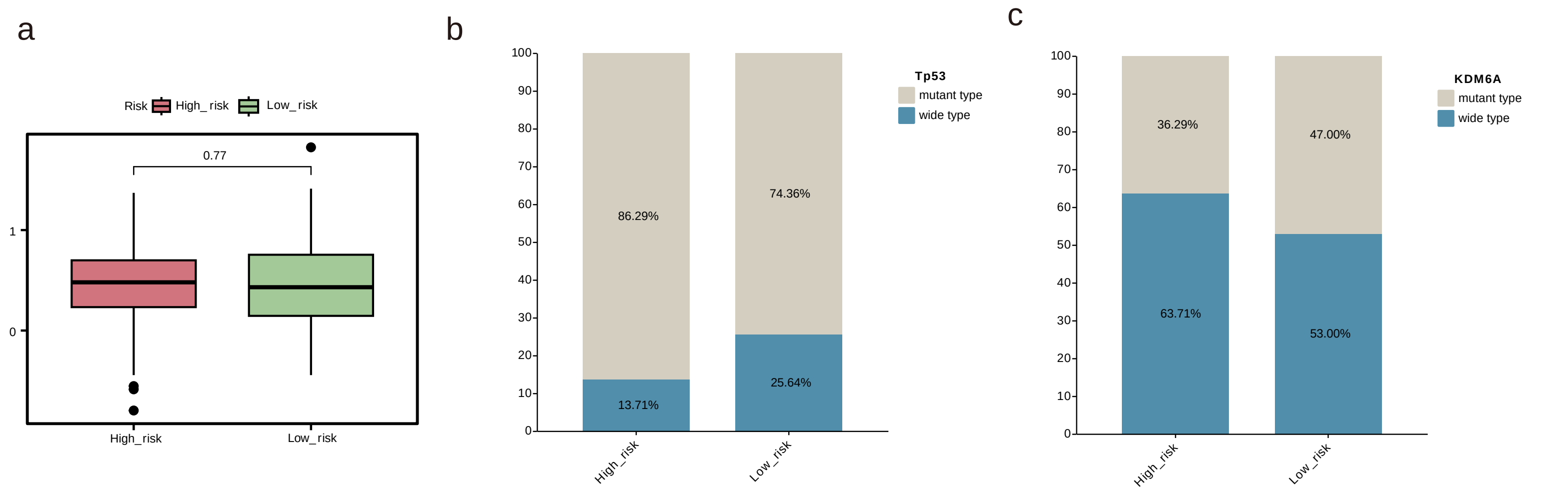
a
Risk High_ risk Low_ risk
0.77
1
0
High_risk
Low_risk
b
Tp53
mutant type
wide type
86.29%
13.71%
74.36%
25.64%
High_risk
Low_risk
c
KDM6A
mutant type
wide type
36.29%
63.71%
47.00%
53.00%
High_risk
Low_risk

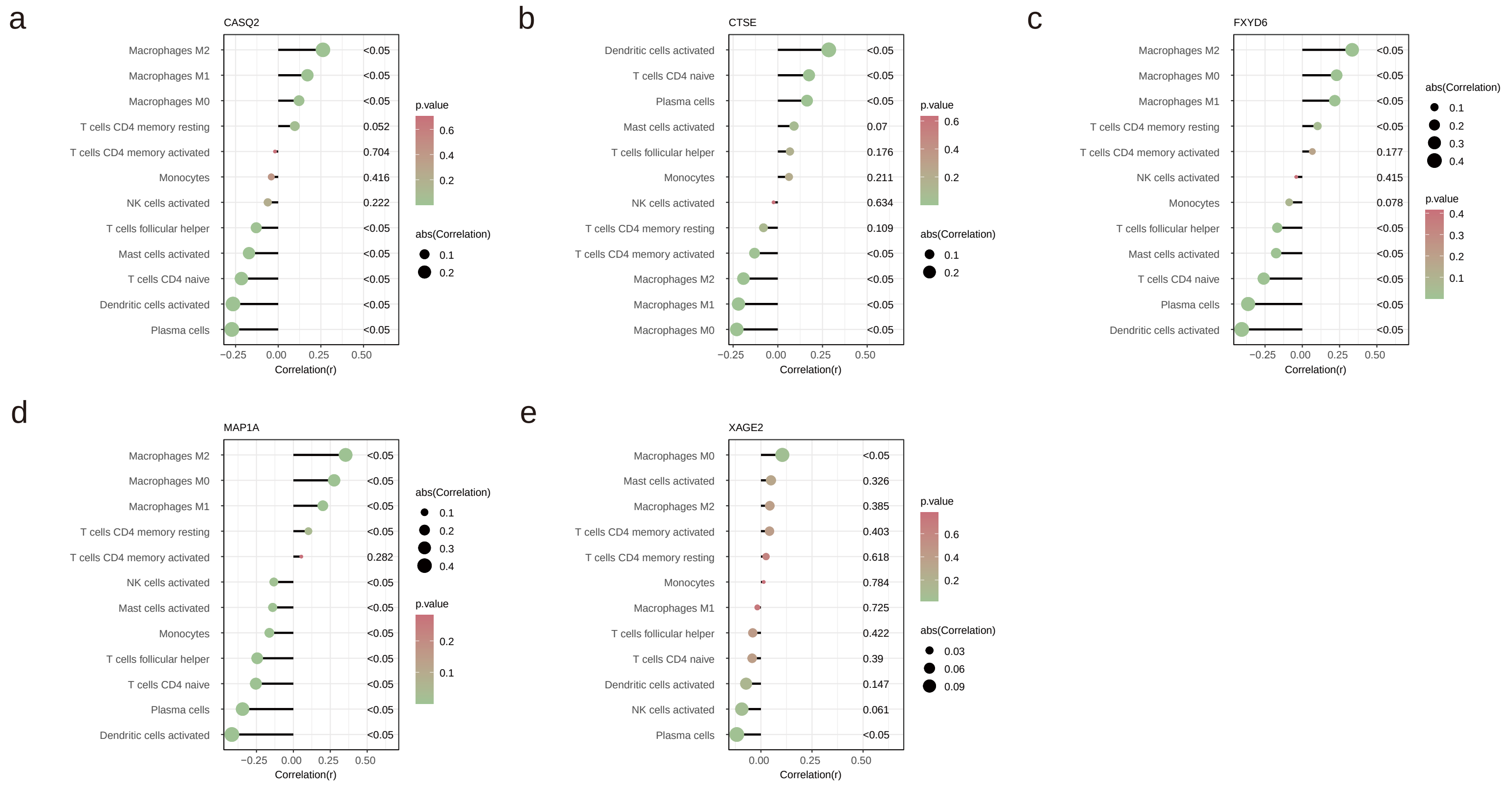
a
CASQ2
Macrophages M2
Macrophages M1
Macrophages M0
T cells CD4 memory resting
T cells CD4 memory activated
Monocytes
NK cells activated
T cells follicular helper
Mast cells activated
T cells CD4 naive
Dendritic cells activated
Plasma cells
<0.05
<0.05
<0.05
0.052
0.704
0.416
0.222
<0.05
<0.05
<0.05
<0.05
<0.05
−0.25 0.00 0.25 0.50
Correlation(r)
p.value
0.6
0.4
0.2
abs(Correlation)
0.1
0.2
b
CTSE
Dendritic cells activated
T cells CD4 naive
Plasma cells
Mast cells activated
T cells follicular helper
Monocytes
NK cells activated
T cells CD4 memory resting
T cells CD4 memory activated
Macrophages M2
Macrophages M1
Macrophages M0
<0.05
<0.05
<0.05
0.07
0.176
0.211
0.634
0.109
<0.05
<0.05
<0.05
<0.05
−0.25 0.00 0.25 0.50
Correlation(r)
p.value
0.6
0.4
0.2
abs(Correlation)
0.1
0.2
c
FXYD6
Macrophages M2
Macrophages M0
Macrophages M1
T cells CD4 memory resting
T cells CD4 memory activated
NK cells activated
Monocytes
T cells follicular helper
Mast cells activated
T cells CD4 naive
Plasma cells
Dendritic cells activated
<0.05
<0.05
<0.05
<0.05
0.177
0.415
0.078
<0.05
<0.05
<0.05
<0.05
<0.05
−0.25 0.00 0.25 0.50
Correlation(r)
abs(Correlation)
0.1
0.2
0.3
0.4
p.value
0.4
0.3
0.2
0.1
d
MAP1A
Macrophages M2
Macrophages M0
Macrophages M1
T cells CD4 memory resting
T cells CD4 memory activated
NK cells activated
Mast cells activated
Monocytes
T cells follicular helper
T cells CD4 naive
Plasma cells
Dendritic cells activated
<0.05
<0.05
<0.05
<0.05
0.282
<0.05
<0.05
<0.05
<0.05
<0.05
<0.05
<0.05
−0.25 0.00 0.25 0.50
Correlation(r)
abs(Correlation)
0.1
0.2
0.3
0.4
p.value
0.2
0.1
e
XAGE2
Macrophages M0
Mast cells activated
Macrophages M2
T cells CD4 memory activated
T cells CD4 memory resting
Monocytes
Macrophages M1
T cells follicular helper
T cells CD4 naive
Dendritic cells activated
NK cells activated
Plasma cells
<0.05
0.326
0.385
0.403
0.618
0.784
0.725
0.422
0.39
0.147
0.061
<0.05
0.00 0.25 0.50
Correlation(r)
p.value
0.6
0.4
0.2
abs(Correlation)
0.03
0.06
0.09
